# Supplementary material for: The emergence of trust under conditions of distrust
Source: Sci Rep. 2026 Apr 3;16:11352. doi: 10.1038/s41598-026-36825-3 (PMC13049019; doi:10.1038/s41598-026-36825-3)
Supplement: Supplementary file 1 — Supplementary Material 1 [file 41598_2026_36825_MOESM1_ESM.docx]

Trust in distrust

**The emergence of trust under conditions of distrust**

**Author:** *anonymized*

**Abstract:** The genesis of trust between strangers on the Internet - without the protective and structuring framework of digital platforms - is a topic that has been little researched so far. This need for research is exploratively approached via an experimental social deduction game, which is intended to make online trust decisions visible under distrustful conditions. A total of 101 participants took part in the nine rounds of the game, whose numerous game and trust decisions were evaluated under distrust-promoting conditions of the game setting. By using this complex experimental setting, various factors influencing trust could be included and it could be shown that trust in the digital space can arise even under persistent distrust and can be observed in the context of a social deduction game.

**Keywords:** trust in strangers; simulation; quantitative social research

**Funding:** No funding.

# Introduction

On the internet, trust between strangers is widespread and often indispensable. At the same time, abuse of trust is made easier by the structural conditions of the internet: dissociative anonymity, the possibility of circumventing sanctions and invisibility all contribute to the fact that the trust that is often necessary in online exchanges is highly vulnerable (Danquah et al., 2022; Danquah et al. 2020; Longe et al. 2012; Suler, 2004).

Trust also appears to arise in inherently distrustful environments, such as in the digital resources exchanged in many online games bypassing the official infrastructure (Fujita et al., 2011; Woo et al., 2011; Chun et al., 2018) or in the trading of items or information via social networks (Lee, 2018). These exchanges require trust, as there is no system in place to ensure compliance with the terms of the contract.

The seemingly uncritical granting of trust on the internet is deliberately exploited in so-called social engineering, whereby individuals gain access to sensitive information and resources through manipulation (Krombholz et al., 2018). The willingness to place trust is exploited by the party who trusts, who is then tricked into performing a security-critical action (Salahdine & Kaabouch, 2019).

While the dynamics of trust and distrust in the analogue world have long been a core subject in many scientific disciplines, including social psychology, sociology and economics, research into these dynamics in the digital space is still in its infancy (Humer, 2020). Although there are studies on various sales or sharing platforms in which trust generation is explained by reputation effects and trust services provided by platform providers (Liu & Tang, 2018; Kuwabara, 2015; Kuwabara, 2021; Diekmann et al., 2009), these do not apply to the examples introduced at the beginning, in which trust is granted between individual actors without platform-side guarantees and security promises. However, it would be too narrow to define such “distrustful conditions” merely by the absence of platform-based controls. Rather, they encompass a heightened perception of risk and uncertainty, minimal or absent institutional safeguards, and the potential for opportunistic or malicious acts with little recourse for victims. Consequently, although missing external control mechanisms certainly play a role, “distrustful conditions” in this article refer more broadly to those interactional and contextual factors that increase the likelihood of exploitation and make the decision to trust particularly precarious.

In previous studies, such situations were recorded through empirical observations, in particular by observing barter transactions on the dark web (Przepiorka et al., 2017; Laferrière & Décary-Hétu, 2023). The dark web lends itself to the investigation of trust under distrustful conditions, as transactions often take place here without moderation by platforms and legal safeguards hardly exist due to the potential illegality of the transactions. The observation of such phenomena not only poses ethical challenges for researchers with regard to the collection of this data, but also practical research challenges: the recording of intentions and motives for granting trust and the weighing of perceived influencing factors are not possible with pure observation and are often limited to recording the result, namely whether trust was granted or not.

To overcome these limitations and empirically record and analyze the giving and taking of trust without the influence of platforms, a method is therefore presented in which trust decisions can be observed within the framework of an online game. The design is based on a real game concept of a social deduction game. When applied in a scientific context, it could enable the observation of the behavior of people who are strangers to each other at the beginning of the game, interact exclusively online in this fixed game environment, and for whom the temporary assignment of trust can - but does not have to - be a relevant strategy to win the game. The dynamics of trust and distrust that develop between players during the game are recorded and evaluated in terms of the decision to grant or withhold trust. Given that this social deduction game has not yet been used in this form to investigate trust, the study takes an exploratory approach to determine its methodological capacity for capturing interpersonal online trust under distrustful conditions and without the influence of digital platforms. The analysis seeks to address the following questions: What kind of game interactions are carried out and are trust advances made at all? In which game constellations does trust become apparent and when is trust more likely to be granted? Against this backdrop, the present study is guided by the overarching three research questions:

- Methodological Approach: How can the social deduction game “Tank Turn Tactics” be designed and administered so that (dis)trust decisions between unfamiliar participants are observable and analyzable?
- Emergence of Trust: Does trust actually develop between players during the game, and how can the formation of trust be empirically measured?
- Validation with Established Measures: How do the trust-related decisions observed in “Tank Turn Tactics” correlate with established short scales of interpersonal trust (e.g., SOEP-Trust, WVS-Trust)?

By answering these questions, the study aims to address an underexplored area in research on digital trust – namely, trust development in unstructured, distrustful online contexts that lack reliable enforcement or reputational safeguards. While much of the existing literature focuses on platform-mediated interactions (e.g., eBay, Airbnb), fewer studies capture organic trust formation among strangers outside such regulated systems. Specifically, the study explores whether the social deduction game setting could reveal observable trust behaviors that do not fully align with self-reported trust measures, thereby potentially challenging the presumed validity of widely used short scales. It further contributes to the broader literature on digital trust by offering a more dynamic, context-rich method of investigating how and why trust emerges – or fails to emerge – in risky online environments.

The initial step involves presenting a working definition of the concept of trust and deriving the prerequisites that must be fulfilled in order to be able to capture trust as defined in this way. Subsequently, the concrete modeling of trust is explained, taking methodological considerations into account. The following chapter presents the social deduction game, which offers a framework for capturing the previously defined concept of trust. Thereafter, the primarily descriptive-static results from the implementation of the game are discussed and concluded in the conclusion, together with the methodological implications and challenges of measuring trust under distrustful conditions.

Trust as decision

In the scientific debate, trust is perceived as a multi-layered and complex construct, the interpretations of which differ according to various theoretical approaches (Rousseau et al., 1998; Cook, 2001; Gambetta, 1988). While the notion of risk is a central element in many definitions of trust and is prominently articulated by M. Deutsch (1962), scholarly discussions on trust have developed a broad spectrum of perspectives. Luhmann (1989) describes trust as a mechanism for reducing social complexity, while Rotter (1980) and Dasgupta (1988) emphasize the role of expectations regarding others’ reliability. Barber (1983) further differentiates between distinct types of expectations in social interactions. In this article, the theoretical framework proposed by Coleman (1982, 1990) will be drawn upon, defining trust as a unilateral transfer of control over resources, actions, or events, inherently linked to risk and future uncertainty. Accordingly, as Coleman (1990, p. 98) notes, “trust involves putting resources in the hands of parties who will use them to their own benefit, to the trustor’s benefit, or both.” Consequently, people’s decisions to trust are based on cost-benefit considerations. From this perspective, the expectations individuals hold regarding others’ behavior under conditions of uncertainty and risk are taken into account: While one may decide internally to trust (e.g., by expecting the other party to act benevolently), Coleman’s (1982, 1990) definition emphasizes the actual transfer of control—an observable action that carries risk. In other words, trust remains abstract until a trusting individual makes a concrete, potentially vulnerable move (e.g., sharing resources or sensitive information). In the game context, such a move may take the form of giving away valuable resources without any assurance of return. Importantly, the decision to trust is made despite tangible disincentives: betraying others not only removes a competitor but grants immediate in-game advantages. This makes any act of trust a striking deviation from rational self-interest and a particularly robust indicator of intentional trust behavior under hostile conditions. By focusing on the observable decision to relinquish control, the present study adopts a perspective from a theory of action, in which trust does not fully materialize unless it is accompanied by an actual, risk-laden step.

The decision to trust, according to Coleman, is not merely a rational calculation of risks and rewards, but a conscious act of engaging with uncertainty. Trust involves an awareness of this uncertainty and a willingness to accept it in a social interaction. Thus, an essential prerequisite for the establishment of trust is awareness on the part of the individual placing it. Empirically, this leads to the problem that in purely observational procedures, this conscious allocation of trust under risk cannot be identified directly or can only be reconstructed retrospectively, namely when a decision has been made for or against the allocation of trust. Thus, while the final decision is observable, the underlying considerations and thought processes remain concealed.

To ensure that participants recognize the potential risks associated with granting trust, a simulation is used in which the cost of trust is explicitly defined. Specifically, this simulation builds on game-theoretically structured trust games (Buskens & Raub, 2004; Raub & Buskens, 2006; Wiens, 2013) and is substantially extended to capture additional complexities. To simulate conditions of distrust, the rules of the game are designed in a way that creates a situation in which the interests of the players are in opposition to one another. The trust decisions that become visible in this way can be observed and analyzed in the light of the dubious benevolence of the trusting players. A further approach to variable control is offered by direct, downstream questioning as to whether and to what extent the observed actions are an expression of trust despite the knowledge of the risk.

The practical application of these conceptual considerations and requirements for trust assessment will be tested in a game-based method, as explained in more detail below.

The modeling of trust

Modeling the phenomenon of trust is challenging due to its complexity. For a long time, trust was measured through surveys, but this is considered insufficient as trust is an experience- and context-dependent phenomenon (Fehr et al., 2002; Glaeser et al., 2000; Uslaner, 2015) and bias effects can lead to a discrepancy between self-disclosure and behavior (Brehm & Savel, 2019). On the other hand, trust is measured by observing behavior under laboratory conditions in elementary trust game. This is a social dilemma in which one person decides whether to give trust and the other decides whether to honor or abuse this trust. This basic design is used in numerous research studies (Raub & Buskens, 2006; Alós-Ferrer & Farolfi, 2019; Camerer, 2003) or is extended according to the research questions, for example to include promises or gifts (Ismayilov & Potters, 2016; Servátka et al., 2011), in order to capture reciprocity and social norms.

Despite its broad application, the trust game is not free from inherent limitations, as a variety of contextual factors are necessarily excluded in the laboratory, which can result in a fundamental bias in the results for a context-specific phenomenon such as trust. In addition, individual preferences and risk or unfairness aversions have an influence on the decision in the game that is difficult to control (Fehr et al., 1993; Fehr & Schmidt, 1999; Chaudhuri & Gangadharan, 2007). There is also disagreement about what is being measured, raising debate over whether these games truly capture trust, leaving open the possibility that factors such as risk aversion, fairness concerns, or social preferences influence the outcome. Supporting this notion, Cox (2004) compared a standard trust game with a variant where the trustee could not reciprocate; the similarity in trustor behavior across conditions suggests that the decision may be driven by social preferences rather than an unambiguous measure of trust (Beck, 2020; Alós-Ferrer & Farolfi, 2019).

These factors, which lead to a distorted or inadequate assessment of trust, can be controlled by means of downstream, standardized surveys. New variations of the trust game also address, for example, honest and dishonest behavior or distrust (Alós-Ferrer & Farolfi, 2019; Macko, 2020; Johnson & Mislin, 2011; Uslaner, 2018). However, with each extension of the elementary trust game, the decision tree also becomes more complex and the number of possible game decisions increases. Nevertheless, there are certain advantages associated with this approach: In practice, many situations in which trust emerges are highly complex and cannot be assessed with complete certainty beforehand. By introducing these additional factors, expanded versions of the trust game can offer significant advantages in capturing the nuanced dynamics of trust formation, even if they inevitably complicate the decision structure.

An additional methodology for the investigation of trust is field observation. The advantage of this method over the elementary trust game is that it has high external validity. However, there are difficulties in recording online phenomena, as it is not clear beforehand whether, when, and where they take place. Furthermore, online privacy settings can completely prevent observation. This is particularly relevant to observations that take place in private conversations or closed communities, where data protection guidelines prohibit the collection of data or where the person being observed simply does not consent to being observed (Evans, Elford, & Wiggins, 2008). Furthermore, it is not possible to reconstruct from the observation whether it was a decision of trust made in awareness of a potential risk, as this awareness cannot be observed. Even if the person is contacted afterwards for a retrospective survey, memory or response distortions can impair the validity of the results (Sudman & Bradburn, 1973; Tourangeau et al., 2000).

The explorative approach presented in the following is intended to combine the strengths of each previously mentioned method (laboratory, field observation, survey) to a game-based method (Slegers et al., 2016) and simulate the complex and situational dynamics of online reality. At the same time, a controllable environment is created for collecting research data on the phenomenon of trust under distrustful conditions.

To this end, participants play a text-based online game that incorporates a variety of game-theoretical elements (Leyton-Brown & Shoham, 2008; Tadelis, 2012; Osborne & Rubinstein, 1994) designed to measure relevant aspects and in a manner where giving trust is highly advantagous, such as decision-making options and information about the decisions of others, interactions between players and a monetary payout for the winner of the game. The acts of trust that can thus be traced in their process of emergence and internal negotiation are to be surveyed and exploratively analyzed in accordance with the methodological approach described below.

## Methodical approach

Capturing trust under distrustful conditions

To capture the phenomenon, a social deduction game is chosen whose interaction decisions are recorded qualitatively and quantitatively. A social deduction game is an informal game in which players pursue opposing goals while retaining the possibility of communicative exchange during the course of play (Hu & Szegedy-Maszak, 2016). Social deduction games therefore focus on social relationships and manipulation between the players. At the same time, trust among the participants is a critical factor in winning these games, as alliances, deception, and credibility play decisive roles. Well-known representatives of this genre include the commercial games Mafia, Among Us or The Werewolves of Bleak Forest. These types of games have previously been studied in terms of the use of trust and distrust-inducing language features and the effects of the game environment on game behavior (Girlea et al., 2016; Zhang et al., 2022; Xiong et al., 2017). Furthermore, trust and distrust and the maintenance of trust in the course of a social deduction game were analyzed (Soylu & Bener, 2018). Social deduction games allow the observation of trust in a hostile environment due to their competitive nature and their conception as an N-person zero-sum game.

In the present experimental setup, the social deduction game is tested without predefined role assignment. There are no fixed roles for trust-giving or trust-taking persons, as is common in conventional simple trust games. This conceptual decision offers several advantages: With a fixed assigned role, authentic emotions and behavior with integrity can only be reconciled if the players can empathize and think their way into the role or are talented actors (Lopes-de-Oliveira & Santos, 2019). In addition, the probability of authentic behavior with spontaneous and flexible interactions is higher, which increases the external validity of the design and allows the interplay of thoughts and behavior of the players in the game situations to be adequately captured. Compared to the simple trust game established in trust research, this provides more flexibility in terms of decision-making options, as each person playing can be both trust-taking and trust-giving at the same time (Rapoport, 2001). In addition, it can be ensured that players are aware that the decision to cooperate or defect with their fellow players is a completely free and therefore potentially influenceable decision.

In order to be able to observe as many decisions of trust and distrust as possible, the game is designed in such a way that defecting behavior is not only worthwhile but can be indispensable for your own advantageous outcome of the game: In order to win, the other game pieces must die. This setup deliberately creates a context in which distrust is structurally encouraged. Players are constantly exposed to incentives that reward elimination over cooperation, and resource transfers provide no direct benefit to the giver. The game thus mirrors environments where trust is highly vulnerable – yet remains a possible, if risky, strategy. This raises the central empirical question: under what conditions, if any, are players still willing to trust, despite the real possibility of betrayal? In this environment of general distrust, trust decisions can be strategically helpful in the course of the game, but they entail a conscious risk for the players, as every trusting person is also a competitor and must be eliminated before the end of the game. The trust decisions that can be observed in this way emerge with the knowledge of the risk of later betrayal that comes with trust. This design deliberately mirrors theoretical models of digital trust dynamics, where individuals must decide to trust despite considerable risks of exploitation and betrayal. By replicating these high-risk conditions in a controlled game environment, the mechanics simulate real-world online interactions in which users continuously weigh the benefits of trust against the potential for defection. The game designed in this way can be understood as an analogy for explaining success in social engineering situations in the real (Internet) world, as it reflects the ambivalence between trust and distrust and enables the dynamics of deception, manipulation and betrayal in social interactions to be depicted and understood. The game aims to depict the phenomenon of placing trust in initially foreign actors on the Internet, who - just as in the game - would have a high expected benefit from defecting. In contrast to the real-world phenomenon, the situational awareness of all game participants will presumably be comparatively high due to the knowledge of the rules of the game, which may mean a restriction of the transfer to reality, but at the same time ensures that trust is granted in awareness of the risk and thus ensures the necessary prerequisite for the underlying definition of trust according to Coleman. In addition, this decision allows far-reaching control of the setting, which proves to be difficult or even impossible if real-world trust situations of online social engineering are only observed and it is not possible to determine whether the actors are aware of their possibly risky use.

Unlike conventional trust games, giving action or life points in this setting provides no direct in-game benefit to the giver. This design ensures that any resource transfer occurs without immediate compensation and is motivated by expectations – or hopes – of future reciprocity or reduced hostility. The absence of a guaranteed reward makes trust especially fragile: players who initially give points may later withdraw cooperation upon realizing there is no tangible payoff, while some may remain willing to trust for purely strategic or social reasons. Contradictory patterns can thus emerge, illustrating how trust can be offered or revoked in an environment where defection is frequently the more profitable choice.

In contrast to many conventional trust games, which involve relatively short and discrete decision points, the game presented here unfolds over a longer period of time, with the duration determined by the players’ decisions. This extended time span enables deeper analysis of how trust dynamics develop, evolve, and potentially collapse, thus going well beyond the narrower, short-term choices typical of simpler trust paradigms.

Data collection and analysis

The process of data collection and analysis in games can present certain challenges (Allen et al., 2024). In order to meet the various requirements for the data to be collected, the game need to be programmed in accordance with the necessary specifications. For a comprehensive approach, various data are collected and analyzed during the game in order to obtain as holistic a picture as possible of the course of the game and emerging trust. A central component is the formal game decisions generated in the game (Appendix 1), which can be freely chosen within the framework of the game rules. Players also have the opportunity to communicate via chat messages (private and global). In addition, all players formulate their thoughts during the game (think-aloud method), which are to be used in the later evaluation as verbal protocols to validate potential trust situations (purposeful, intentional and conscious behavior according to the previous definition of trust, see Appendix 2.

Accordingly, the data corpus consists of the game decisions, the communication between the players and the verbal protocols. In the present study, the game decisions and the verbal protocols were analyzed and used to validate the results, as discussed in the following two subsections.

Game rules and game decisions

Beginning with a brief, purely descriptive description of the rules of the game, their rationale and effects are supplemented below. The rules of the game are taken from the unpublished game prototype “Tank Turn Tactics” designed by Halfbrick Studios (Luke Muscat, 2013), specified and adapted to the scientific-experimental context. All participants are each given a game piece, which is randomly placed on a square of the game. The playing field is set up like a chessboard, the size of which is scaled depending on the initial number of game pieces, so that the game pieces are at similar distances from each other to ensure comparable starting conditions between the different game sessions (Img. 1).


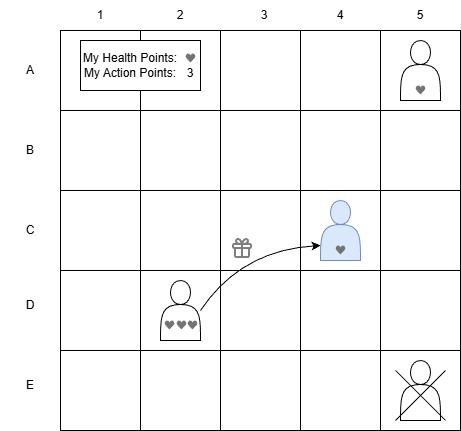


Img. 1: Exemplary board view, showing an active game piece (light blue) receiving a gift from another game piece within reach. Based on the illustration from Fehlhaber & El-Awad, 2024.

Each character has three values: life points, action points and range. The life points must not fall to 0, otherwise the character is considered dead. They are symbolized by hearts on the character and are visible to all players. The action points are required to carry out actions (movement, actions directed at yourself, resource transfer or attack). Actions can be carried out at any time as long as enough action points are available. Action points can be acquired in two ways: Either by waiting for the daily and automatic allocation of action points or by killing other game characters, whose collected action points become the property of their murderer. The participants are only aware of their own action points, so they do not have complete certainty about the actions of the other characters. Moving to an adjacent square costs one action point, as does attacking a character, which will cost them one life point. The transfer of resources takes place 1:1, i.e. if a character gives away an action point or a life point, this costs them an action point or a life point respectively. The range determines the radius in which a character can carry out transfers and attacks. At the start of the game, this radius is two spaces, but can be increased during the course of the game by taking an action (“extend range”). The aim of the game is to have a pawn with at least one life point, while all other pawns have no more life points.

| **initial situation**  HP (health points): 3  AP (action points) :0  Range (action radius) :2 | **effects per day** (timing may vary)  ⋅ all players with 0 HP can vote to haunt a player  ⋅ all players who are not haunted receive 1 AP  ⋅ a heart is dropped at a random tile; a player moving on the tile gains 1 HP | **effects on defeat** (reduction of a players HP to 0)  ⋅ the AP of the defeated player goes to the one who conquered him/her  ⋅ the defeated player joins the ghosts and can vote to haunt | **victory condition**  all player but one have 0 HP |
| --- | --- | --- | --- |
| \| Possible Action \| Cost \| Target \| \| --- \| --- \| --- \| \| move 1 square \| 1 AP \| self \| \| shoot (reduce the HP of player in range by 1) \| 1 AP \| another player \| \| add a heart \| 3 AP \| self \| \| upgrade range \| 3 AP \| self \| \| revive \| 1 HP \| another defeated player \| \| transfer 1 AP \| 1 AP \| another player \| | | | |

**Tab. 1:** Cheat Sheet of Game Rules. *Notes:* *HP: Health Points, AP: Action Points. Source: Fehlhaber & El-Awad 2024.*

The life and action points can be transferred and thus given away. In the game framework designed in this way, cooperation and trust decisions as well as the choice of transaction partner are completely optional, unlike in the conventional experimental trust game.

The game is played in two variations: “Standard” and ‘Quick’. The rules for both variants are identical except for the allocation of action points. These are awarded twice a day in the standard rounds, but at a higher frequency of hourly to minutely in the quick rounds. This variation makes it possible to examine the time factor as an independent variable and to analyze how time pressure influences the development of trust under distrustful conditions. Demanding trust while simultaneously creating time pressure for the trust giver is a typical strategy in social engineering (Fountouki et al., 2018; Hadnagy, 2018).

The most important rules of the game and their intended impact on the game are discussed below. The complete set of rules as handed out to the participants, but without explanations, can be found in Appendix 1^^[[1]](#footnote-1)^^.

At the beginning, the playing pieces are randomly placed on the playing field. Each has three life points and one action point. In the standard rounds, two action points are given to each player at a random time each day. In the quick rounds, one or more action point(s) are awarded per hour at a random time. The standard rounds give the players more time for communication, planning and execution of actions, while the quick rounds increase the dynamics of the game, as the players have more action points more quickly, which increases the pressure to act and the need for spontaneous decisions. The variability of the timing in the allocation of action points is intended to motivate players to keep checking in on the game and at the same time avoid agglomerations of action point expenditure at a known and fixed time, giving faster players (in terms of reaction time) an advantage in the game.

The possible actions are explained below, along with an explanation of the possible effects of these rules.

- Movement: Moving your own playing piece to another square can be used as a strategic maneuver for better positioning, to escape, to prepare an attack or to prepare a trusting interaction and costs one action point per square. If a pawn is moved to a square on which another pawn is standing, it is moved one square and another action point is deducted from the person responsible.
- Self-directed action: For every 3 action points, characters can permanently increase their own range by one space or regenerate one life point. This can create an incentive for interaction between the players, for example because a pawn that can increase its range several times has a significantly greater strategic advantage than several pawns that increase their range by 1 each. Furthermore, the cost of increasing a life point, for example, is proportionally more expensive than making an agreement with other players. These game actions can offer an alternative to interactions with other players, but are correspondingly expensive.
- Resource transfer: Players can transfer life or action points to another player within range of their character. This game rule makes it possible to give and take trust. Giving valuable life and action points is risky, as the trust-giving players cannot know whether their trust will be rewarded or whether the gifted players will use their improved resources to weaken the giving players or get rid of them. If a dead character is given a life point, it is revived. This gives them another chance to actively participate in the game and possibly even win, while the person giving the gift is weaker as they have lost (at least) one of their own life points.
- Attack: To win the game, the other characters must die by being attacked. Each attack costs one action point and subtracts one life from the attacked character. If the playing piece has 0 life, it is considered dead, but remains on the playing field for everyone else to see. In addition to winning the game, every destruction of other playing pieces promises the additional incentive of acquiring their action points. This not only compensates for the relatively high expenditure of action points for the attacks, but also creates an additional reward that promises more freedom of action in the future (through more available action points).

Dead characters can no longer carry out regular actions. Instead, they form a jury and can vote in secret each day on which living character should be “haunted”: The character that receives the most votes is considered haunted and will not receive any action points that day. This allows dead players to take revenge. This game rule not only allows you to observe whether cooperative actions by the dead characters are taking place, but also sanctions past behavior by (still) living characters (“shadows of the past”).

The game ends when there is only one living pawn left, which is awarded 1st place and wins the game. The last and next-to-last living pieces on the playing field are awarded 2nd and 3rd place respectively, with different real money values associated with the placement.

The outlined rules of the game create an environment that enables the participants to control resources. Passing on valuable resources can be seen as a sign of trust in this game environment. The participants are not forced to provide proof of trust and make trust decisions in order to win the game.

Translated into the actions of the observational study,, these rules result in the following possibilities for game interactions: Attack, own movement, cause movement of a fellow player, regenerate own life, own range increase, give away life (revive if necessary), give away action points and haunt (prevent someone else from getting daily action points).

Clusters were established by mapping each game action to one of three pre-defined categories—friendly, neutral, or hostile (e.g., an attack is classified as hostile, while a gift is classified as friendly, see Tab. 2). This method simplifies the analysis by reducing the complexity of numerous specific actions into clear, overarching behavioral types, thereby enhancing clarity and facilitating systematic comparisons across sessions for later analysis.

| Type of action | Classification |
| --- | --- |
| (attempted) attack | hostile |
| haunt | hostile |
| (attempted) regenerate own life | neutral |
| (attempted) own range increase | neutral |
| (attempted) transfer life points | friendly |
| (attempted) transfer action points | friendly |
| end haunt | friendly |

Tab. 2: Game interactions and classification into one of the three interaction types (hostile, neutral, friendly)

If respondents attempted to carry out game actions but failed due to a lack of action points, for example, or contradicted the rules of the game, this was noted for the analysis with the prefix “attempted”. Although these actions were not carried out, they still indicate the respondents’ intentions and were therefore recorded separately. Movements were not assigned an analysis attribute, as although they were necessary to play the game, they always took place in preparation for a friendly or hostile game interaction and do not themselves allow a clear classification. Accordingly, only the formally unambiguous results from the movements are analyzed, for example an attack resulting from a movement or the giving away of life by moving into the range of a fellow player.

To further systematize the analysis of in-game behavior, the concept of game tactics is introduced. Recognizing the complexity of individual game actions, recurring patterns were clustered into distinct tactical groups. Specifically, these clusters were formed based on the type of action – categorized as "Offensive," "Self-oriented," and "Cooperative" – and their relative frequency within each game session (see Table 4). This tactical classification simplifies the analysis by reducing a diverse set of game actions into comprehensible groups and provides a structured framework for linking these behaviors to game outcomes, such as survival time (see Table 5).

Verbal protocols

The method of verbal protocols can look back on a long research tradition and has been increasingly used in various fields of application since the cognitive turn and thus a departure from behaviorism (Danziger, 1980). Verbal protocols make it possible to gain insights into people's thoughts and feelings (Konrad, 2020; Ericsson & Simon, 1998; Ericsson & Simon, 1993). This is achieved by asking participants to think aloud while analyzing or solving a problem (Someren et al., 1994; Nisbett & Wilson, 1977). By verbalizing thoughts in this way, not only cognitive processes, but also themes and attitudes or their social construction can be understood when the resulting data is analyzed discursively or narratively (Edwards et al., 2012). While recognizing that the verbal utterances can inevitably be colored by cognitive biases (Yang, 2003; Steen, 1994), the value of this method is nevertheless seen in the possibility of being able to understand subjective evaluations of the participants.

The written statements in the verbal protocols about the assessments of other participants in the game were recorded using a summarized content analysis (Mayring, 2015), following a deductive-inductive strategy. Building on conceptual definitions of trust and mistrust (Endress, 2015) as overarching, predefined categories, these categories were refined through close examination of the data, resulting in subdimensions such as “high trust,” “low trust,” “high mistrust,” and “low mistrust”. Instances were also identified in which a trust interaction did not occur, for example due to “unintentionality” (e.g., a resource transfer made inadvertently by entering an action command too quickly) or “testing” (e.g., initially trying out how resource transfer works). Interactions coded as “unintentionality” or “testing” were not evaluated as acts of trust. Finally, the qualitative analysis was conducted using the four-eyes principle for communicative validation of the results (Meyer, 2018; Przyborski & Wohlrab-Sahr, 2021).

## For the present study, only those categories were used that allowed the validation of trust interactions, i.e. “unintentionality” and “testing”. This makes it possible to assess whether an action initially classified as friendly (transfer of action points or life points) also corresponds to a conscious and potentially risky trust interaction according to the theoretical basis. The other qualitative categories of trust feelings have not yet been included in the analysis strategy but could provide deeper insights into the genesis of these feelings in further research.

Capturing evidence of trust and trust decisions

Evidence of trust and subsequently trust decisions are recorded using a formal definition, according to which the trust-giving person A, influenced by the trust-taking person B, either uses at least one action point for a cooperative action with B or A gives away action or life points to B. Such a constellation is then considered evidence of trust.

Formally, this proof of trust should be defined as:

$$A influenced_{by\left( B \right)}\wedge\left( A used_{Actionpoint for cooperative action with \left( B \right)}\vee A transferred_{Actionpoint} \vee A transferred_{Lifepoint} \right)\supset A \mathrm{demonstrated}_{trust(B)}$$

The verbal protocols are used to validate whether the proof of trust was the result of a decision to place trust or an oversight. Only then is a transfer of resources or cooperation classified as a conscious decision to trust. A decision for trust should be assumed if the verbal protocols do not imply otherwise (e.g. an unintentional game action due to carelessness or an initial testing of game commands).

Accordingly, a trust decision is formally defined as:

$$A decides_{to trust\left( B \right)}\wedge A demonstrat{es}_{trust\left( B \right)}\supset A trusts \left( B \right)$$

The result is captured in binary (trust or no trust) and used to further analyze trust and trust decisions under distrustful conditions.

# Study

## Relationship to Prior Work

The gameplay material examined in this study has also been used in a previously published analysis that approached the sessions from a network-analytic perspective (Fehlhaber & El-Awad, 2024). In contrast, the present manuscript addresses a different research aim: it evaluates the suitability of the game as a methodological tool for observing trust-related behaviour under distrustful conditions and focuses on individual decisions, behavioural patterns, and their interpretation rather than on network structures. Both studies therefore draw from the same empirical source but examine fundamentally different analytical questions.

## Study Setup

The entire study and the preparations are carried out online. Participants take part in an experimental game using an internet-enabled device and are asked to verbalize their thoughts during the study. This verbalization of thoughts and experiences is practiced individually with the participants via a video conferencing system prior to the actual study. Their decisions during the game and the qualitative data are collated and subsequently analyzed.

The test procedure is structured as follows: After obtaining a declaration of consent to participate, assurances that the data will be collected in accordance with data protection regulations and that the data generated in the game will be used for research purposes, the following instructions are read out to the participants: “Please say everything that comes to your mind and goes through your head [...]. It is important that you do not try to explain or structure what you are doing. Just imagine that you are alone in the room and talking to yourself” (Heine & Schramm, 2007, p. 178). Subsequently, the verbalization of thoughts is learned and practiced in a warm-up phase in order to prevent potential difficulties during the main study (Ericsson & Simon, 1998). In the learning and warm-up phase, the participants play a digital version of the board game Trouble, whose mirror rules are widely known or at least very easy to learn in English-speaking countries. Participants are then offered the rules of the game, which are also stored as a document for the entire time before and during the study. The e-mail address of the study leader is also included in the filing system so that participants can contact him/her at any time if necessary.

At the end of the practice and warm-up phase, participants have the opportunity to ask questions. They then receive their access code for the experimental game and are asked to log in once and familiarize themselves with the functions (game board and chat rooms) in order to rule out technical difficulties on the part of the participants and their end devices. They are then free to decide how often they log in and play during the game phases (daily from 2pm to 10pm). Only one login every 48 hours is mandatory in order not to be excluded from the study and thus permanently removed from the playing field. The game can be terminated at any time and without giving reasons. The game ends when there is only one person left. The expense allowance or prize money will be paid out no later than four working days after the end of the respective game, ensuring both the neutrality of game behavior by avoiding immediate financial incentives and proper logistical handling of payouts.

Immediately after the end of the game, a follow-up survey is conducted with the participants. For this purpose, they are presented with a scale on trust in strangers (Appendix 3: SOEP-Trust according to Naef & Schupp, 2009, an improvement of the General Social Survey (GSS) and World Values Surveys (WVS), reliability coefficient ρ of 0.81 and Cronbach's alpha of 0.66). Both the SOEP Trust Scale and the single item GSS/WVS^^[[2]](#footnote-2)^^ are used for the subsequent evaluation of self-reported trust in strangers.

Game environment

For the study, a specially set up Discord server was used as an audio and video conferencing environment, which was expanded to include self-programmed additional and game functions. A file repository with the rules of the game, chat functions for individual and group conversations with other players and a separate sub-area for the game were set up. The game board and the positions of all players can be called up in this area, and actions can be carried out during the game. Players can also view their own action points. The programming of the game rules was carried out by the author.

Whenever the participants log on to the server, the study administrator is notified by a script and is in a single video room with the relevant person. While they participate with audio and video for the entire duration of their login, the study administrator is not visible via video and only switches on the microphone when necessary, for example to remind the participants to think aloud. This also makes it easy for participants to log in at the same time and enables simultaneous supervision during the game. Importantly, participants do not have video or audio communication with each other, relying instead on text-based chat for both individual and group conversations. This restriction reduces the potential for building rapport through nonverbal or auditory cues - an element that may influence how trust and distrust develop during the game. Nonetheless, participants can freely access other areas on the server - such as the game interface and chat rooms - without interrupting or disabling any audio or video connections with the study administrator.

## Each session is recorded on the server so that a retrospective evaluation of the verbal protocols can be carried out even if several participants are playing at the same time. The participants were informed of this at the beginning of the study, and a corresponding note reminds them of the recordings of text, audio and video during each session.

Pretest of the study

To check the survey instruments, a pre-test was carried out in advance with six participants. The majority of participants found the Trouble game exercise to be too long. This task was subsequently modified to familiarize participants with the verbal protocols so that they only played until they felt confident with the method. The simultaneity of playing and verbalizing was described by most of the participants as challenging, especially at the beginning, but after some time the writing of verbal protocols became increasingly routine.

The technical implementation of the study itself went smoothly. Group chats were not used by the participants during the pre-test, which is why the function was explicitly pointed out in the subsequent sessions. All but one of the participants logged into the game at least once a day, even after dying. The pre-test game lasted 7 days. In the post-test survey, the game experience was described as motivating and captivating, with some participants reporting a high level of emotional involvement that they had experienced during the game.

Sample

The sample was recruited via the Reddit platform using current best practices for online studies (Kühne & Zindel, 2020; Ho, 2020; Stern et al., 2017; Ford et al., 2019). This sample was chosen because it represents a diverse group of internet-savvy users recruited from Reddit, who are familiar with digital communication even if they do not necessarily have prior experience with experimental settings. A total of 101 people took part in the nine game sessions. Basic demographic information was collected during recruitment, albeit based on unverified self-reports, but participants were explicitly instructed not to disclose any personal details during the game interactions themselves, in order to avoid influencing trust-related behaviour within the sessions. Three of these sessions were conducted under adapted game rules: Action points were awarded every hour, every minute and every two minutes, instead of twice a day as in the standard game.

## Analysis and discussion of the results

This chapter provides an integrated overview of the analysis and results. It begins with a concise descriptive overview of key session metrics such as duration, participant numbers, and the distribution of friendly, neutral, and hostile interactions. The subsequent section examines game tactics, exploring how different strategic behaviors—ranging from offensive and self-oriented to cooperative—relate to performance outcomes and the formation of trust.

Further, the chapter explores how variations in playthrough dynamics lead to unique interaction patterns and trust decisions, even under similar initial conditions. The impact of time pressure is also considered by comparing standard and accelerated sessions, revealing observable trends in interaction attributes and trust behavior under different temporal constraints.

Additional insights are provided on the interplay between hostile actions and trust decisions, highlighting how early hostility can undermine emerging trust while attempts at reconciliation sometimes occur thereafter. Finally, the diversification and depth of trust are analyzed, showing how pre-existing trust levels and overall game activity influence both the spread and intensity of trust relationships.

The chapter concludes with a discussion of limitations, addressing content-related and conceptual challenges.

Descriptive analysis

The standard sessions covered total play durations between 32:04 and 2278:41 hours. The quick sessions lasted 65:07 hours (hourly action points), 19:01 hours (two-minute action points), and 19 minutes (minute action points). The strong variation in session duration (see Tab. 3) is due to the coexistence of two structurally different session types: very short quick sessions, which were implemented as a separate experimental format, and full-length sessions. The full sessions also differed substantially in group activity, with some groups interacting continuously and others ending early due to eliminations or low engagement. As a result, overall session length reflects emergent group dynamics rather than a controlled parameter.

An average of 11 participants (SD = 5.3) took part in each session. Across all sessions, there was an average of 84.6 game interactions (SD = 53.16) that could be assigned to clear attributes (hostile, neutral, friendly). If the quick sessions are excluded, the average number of assignable actions increases to 110.5 (SD = 45.17) for the standard sessions. In the quick sessions, an average of 33 interactions with reduced variance (SD = 14.1) can be determined.

| Session | Number of players | Game interactions | Playing time (in hours:minutes) |
| --- | --- | --- | --- |
| 1 | 8 | 67 | 32:04 |
| 2 | 12 | 71 | 431:25 |
| 3 | 15 | 120 | 946:37 |
| 4 | 17 | 129 | 83:12 |
| 5 | 15 | 187 | 510:22 |
| 6 | 18 | 89 | 2278:41 |
| Q1 | 7 | 46 | 65:07 |
| Q2 | 5 | 18 | 19:01 |
| Q3 | 4 | 35 | 0:19 |

Tab. 3: Overview of game sessions, number of players, game interaction and game duration.

Due to the aforementioned different number of total interactions in the individual sessions, a comparison between the sessions is only possible by means of normalization. By adding the percentage values, it becomes clear that the ratio between hostile, neutral and friendly interactions is similar across all game sessions. There are a few notable exceptions, such as in quick session S2, in which no friendly interactions were carried out, or in standard session 5 and quick session S3, in which below-average or no neutral interactions were found. In total, 65.5% hostile, 25.1% friendly and 9.4% neutral game interactions were observed (Fig. 1).

#
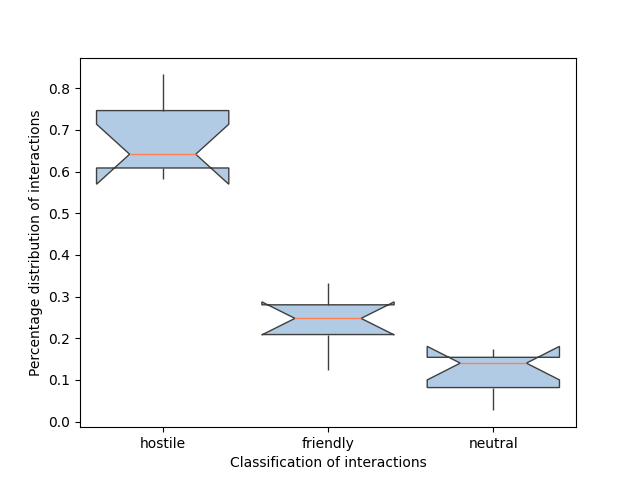


Fig. 1: Distribution of hostile, friendly and neutral interactions in the nine game sessions

Together with the verbal protocols, each game interaction was checked to determine whether it involved trust decisions that met the previously defined trust assignment criteria. Only then was a friendly game interaction classified as a trust interaction (Fig. 2).


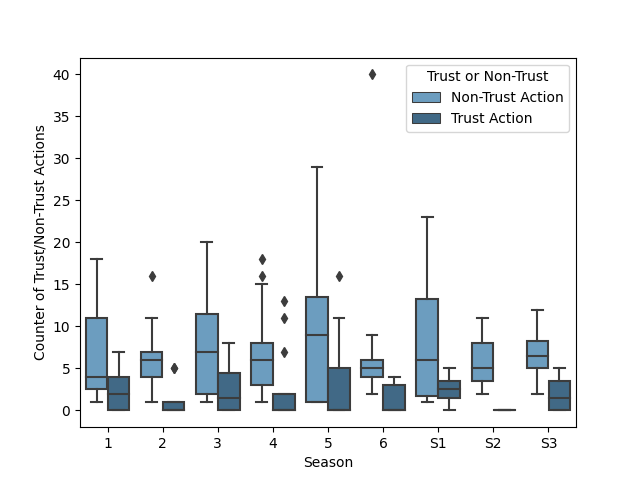


Fig. 2: Trust and non-trust decisions in the various sessions (standard session: 1-6, session: S1-S3)

A total of 173 trust decisions were observed in the nine game sessions. An average of 2.3 trust decisions (SD = 3.4) were made per game. As a percentage of the total interactions, there is a difference between the standard sessions and the quick sessions: While between 9.2% and 19.7% (M = 14.3%) trust interactions were realized in the former, these are between 3.9% and 5.2% (M = 4.8%) in the quick sessions.

Moreover, an investigation will be conducted to ascertain which respondents exhibited trust in the game and whether this was feasible with the conventional scales (SOEP-Trust, Trust in Strangers as an extension of the WVS question, and the WVS question separately due to its high prevalence). Using the multi-item scale for interpersonal trust, specifically for trust in strangers (SOEP-Trust, Appendix 3), a value was determined for each participant based on the mean index. Analogous to the four 4-point Likert items, this can take on values from 1 to 4. The higher the value, the more trustworthy the respondents consider a stranger to be. For the two questions where the scale was reversed, an inversion was carried out. The average value of all players was 2.1 (SD = 0,4, Q_1_ = 1,8, Q_3_ = 2,5) with a minimum value of 1.5 and a maximum value of 3.0. This shows that the sample tends to be somewhat distrustful of strangers. The first question of the multi-item scale, which is also the item of the World Value Survey for recording trust, is just above the average of the SOEP Trust Scale (determined via the mean value index) with an average of 2.2 (SD = 0,9, Q_1_ = 1,5; Q_3_ = 3,0).

Game tactics and consequences in the game

The game “Tank Turn Tactics” can be seen as a microcosm in which trust is examined under conditions of uncertainty and distrust. Reports from the game’s creators indicate that the title has even been banned in their workplace due to the level of interpersonal hostility it generated. Although anecdotal, this observation illustrates the highly adversarial nature of the environment and supports the premise that the game reliably evokes distrustful conditions. It therefore provides additional justification for studying trust in this context, as the setting exposes participants to precisely the kind of social pressures in which trust becomes consequential.

Six clusters were formed to summarize the different game tactics. These were formed based on the type of action (“Offensive”, “Self-oriented”, “Cooperative”) and their relative frequency in the respective game session (Table 4).

| Cluster name/number | Offensive | Self-related | Coope- rative | Cluster Description |
| --- | --- | --- | --- | --- |
| 0 | None | None | None | No action |
| 1 | Existent | None | None | Only offensive |
| 2 | x* | Existent | None | Self-orientated without cooperation |
| 3 | x* | None | Existent | No self-oriented actions and little cooperation |
| 4 | x* | Existent | Existent | Few self-oriented actions and little cooperation |
| 5 | x* | x* | Predominant | Cooperation dominates |

| Tactics | Related Action(s) |
| --- | --- |
| Offensive | Attack, haunt |
| Self-related | Increase range, heal yourself |
| Coope- rative | Give away AP, give away HP, end haunt |

Tab. 4: Summary of the players' behavior regarding game tactics. In this context, the use of an asterisk indicates a variable, unspecified number of actions.

The strategies developed in this way were initially set in relation to the survival time (Table 5) and thus in direct dependence on the success of the game. Due to the expected frequency of less than 5 in some of the observations, Fisher's exact test was used instead of the regular chi-square test.

For the “Cooperative” tactic, the significance test just misses the alpha level of .05 with p = .057. For the “Offensive” and “Self-oriented” tactic, on the other hand, there is a highly significant correlation with medium effect size (for the “Offensive” tactic) and high effect size (for the “Self-oriented” tactic) between the frequency of use of the tactics and the probability of survival. This suggests that the patterns observed in the game strategies and their effects on success in the game are probably not random but are potentially due to the dynamics of trust and distrust.

| Survival time | cooperative tactics | | | offensive tactics | | | self-oriented tactics | | |
| --- | --- | --- | --- | --- | --- | --- | --- | --- | --- |
|  | predominantly | only few occasions | none | predominantly | only few occasions | none | predominantly | only few occasions | none |
| 1st tercile | 1 | 6 | 24 | 1 | 17 | 13 | 0 | 0 | 31 |
| 2nd tercile | 3 | 8 | 23 | 0 | 24 | 10 | 0 | 8 | 26 |
| 3rd tercile | 8 | 7 | 12 | 4 | 18 | 5 | 0 | 15 | 12 |
| last survivor | 2 | 3 | 4 | 5 | 3 | 1 | 3 | 2 | 4 |
|  | p-value = 0.0569  Cramer's V = 0.2461 | | | p-value = 0.0000  Cramer's V = 0.3865 | | | p-value = 0.0000  Cramer's V = 0.5264 | | |

Tab. 5: Contingency tables for cooperative, offensive and self-oriented tactics.

In addition to influencing the duration of survival and thus the success of the game, it should be noted that actions classified as cooperative occur in several strategies: 14 players can be found in cluster 3 (some teamwork, no self-interest) and 10 players in cluster 4 (some teamwork, with self-interest). For cluster 5 (focus on teamwork), there are a further 14 players who have chosen this tactic. On the other hand, there are 63 players who did not perform any actions (25), focused on offensive actions (26) or primarily performed selfish actions directed at their own character (12). An appropriate heterogeneity of strategies and, above all, alternatives to the allocation of trust are visible here and in the effect on the duration of survival.

In addition, when looking at the individual sessions and the abstracted strategies used by the players, certain agglomerations of game strategies become apparent: If a relatively large number of characters are killed in one session before they can perform their own actions (cluster 0), then at least one more alliance is formed in addition to a frequently existing alliance of allied characters; players overcome themselves in the face of several cooperatively playing characters to trust each other and form a power counterbalance. Certain copycat effects can also be observed in the self-referential actions, with players copying this tactic more often if it is used in a run-through and largely ignoring offensive and cooperative strategies.

A logistic regression was conducted as a robustness check to complement the contingency tables in Table 5. When modelling the probability of reaching the highest survival category (“high”), cooperative tactics served as the reference category. Players with no clear tactic (cluster 0) showed substantially lower odds of high survival (OR = 0.10, p = .005), as did offensively oriented players (OR = 0.21, p = .015). Self-related tactics pointed in the same direction (OR = 0.54) but did not reach statistical significance (p = .43). These analyses support the conclusion that cooperative tactics are most conducive to longer survival in the game.

Assuming that trust does not emerge in isolation from contextual and social conditions, this observation is particularly interesting as it reveals replication strategies that manifest themselves under the distrustful conditions of the game.

Influence of the playthrough

Despite identical or at least similar initial conditions, the courses of the games developed in different ways. Not only is the time parameter (minimum standard session = 32 hours, maximum standard session = 2278.4 hours) characterized by a high degree of variance; the frequency of interactions and the dynamics between the participants were also very different in the various sessions. Before a comparison of the standard and quick sessions is made at a later point, it will first be examined within the standard sessions whether there are significant differences between the game sessions in the quantitative parameters (game interactions and trust decisions) of game and trust interactions.

To test whether there are systematic correlations or deviations between the game sessions, hostile, neutral and friendly interactions as well as trust actions in the standard sessions were represented by contingency tables. Independence is tested using the sum of the squared, corrected standardized residuals (Pearson's χ²), i.e. the deviation of the observed values from the estimated values.

Interactions

In the chi-square analysis, the null hypothesis of independence—that the type of interaction (friendly, hostile, neutral) is independent of the different playthroughs—was tested. The analysis revealed a significant difference between the observed and expected frequencies (χ²(16, N = 762) = 46.72, p < .001), leading to rejection of the null hypothesis at α = .05. The calculated effect size, measured by Cramer's V, is V = 0.175, which indicates a small to medium magnitude.

The divergent social dynamics manifested in each session could explain these differences, as each group developed specific interaction patterns. The game design seems sensitive to divergent social interactions and is able to capture different patterns of behavior. However, replicability of results could be challenging as the unique social dynamics of each group could affect the consistency of behavioral patterns between sessions. However, the aforementioned challenges are typical for observational studies, in which the situational variability of human behavioral patterns often leads to difficulties in replicability.

Acts of trust

The chi-square analysis of trust actions revealed no significant difference between observed and expected frequencies (χ²(8, N = 762) = 14.07, p = .070), indicating that the type of trust actions is not systematically linked to the specific conditions of each game session. In contrast to other interaction types that vary significantly with session dynamics, trust decisions remain relatively stable, suggesting that they are governed more by individual and situational nuances than by the structural parameters of the game. –This pattern supports the view that trust is an inherently complex, context-specific phenomenon which cannot simply be predicted or replicated by structural conditions. In the real world, trust decisions are often unpredictable and highly dependent on individual and situational factors. Therefore, the ability of the game to reflect this unpredictability can be seen as a strength in terms of external validity.

The influence of time pressure

In the following, the extent to which the time pressure generated by the adapted game rules has an influence on the type of game interactions will be examined. Hence, the attributes of the interactions (friendly, neutral, hostile) as well as the trust actions of the standard sessions are compared with the quick sessions. For this purpose, the (non-parametric) Fisher exact test is used, which has no requirements for the number of cases and is therefore also suitable for a small number of observations. The data were transformed as 2x3 and 2x2 contingency tables respectively in order to carry out the comparison between the two groups (standard/fast). Neither for the attributes of the interactions (p = .26) nor for the acts of trust (p = .303) could a significant correlation be observed between the two experimental groups. As a robustness check, the comparison between standard and quick sessions was replicated using logistic regression models at the level of individual interactions, with a binary indicator for trust actions as the dependent variable and a dummy for quick sessions as the main predictor, while controlling for session-level clustering. These models confirmed the descriptive findings: the quick-session indicator did not reach conventional significance levels, and the estimated effect sizes were small. Thus, the observed differences in proportions between standard and quick sessions are not statistically robust.

Hostile interactions before or after an act of trust

A QAP regression (Quadratic Assignment Procedure) is used to investigate a correlation between the interactions between two players (Appendix 4), as the network-like structure of the data makes a normal regression unsuitable.

In three of the nine sessions, highly significant effects were found between trust interaction and hostile interaction (*session 2:* β = 0.28, p = .001, *session 3:* β = 0.25, p = .0002, *session 4:* β = 0.21, p = .0004). In these game sessions, constellations were found in which two players formed a close alliance and transferred resources and helped each other until shortly before the end of the game. In two of these three rounds, the participants expressed their intention to defect in the last third of the game. The respondent who defected first by attacking the trusted partner was subsequently afflicted in all cases without exception. The relationship of trust seemed to be irrevocably destroyed, even if the defecting person had previously provided trusting services (e.g. by resuscitation). In other constellations, there are players who avoided hostile interactions after receiving an advance of trust or tried to (re)establish the relationship with a gift of trust after a hostile interaction.

Despite these findings, it should be noted that these dynamics between hostile behavior and trust could not be proven, at least statistically, in at least two thirds of the game sessions.

Diversification of trust and depth of trust

In the following, acts of trust will be modeled as a dependent variable. On the one hand, the trust dispersion, which describes the number of trust recipients that a trust-giving person has engaged with during the game, is to be examined. Secondly, the depth of trust is to be explained, which describes the intensity of one (or more) relationship(s) with the person receiving trust.

For the analysis, two generalized negative binomial models were created to avoid potentially undesirable overdispersion (Hilbe, 2011) with the dependent variables trust dispersion (number of different alters trusted) and trust depth (number of trust actions toward a given alter). As predictors, the player’s own non-trust actions, the total number of their actions in the session, the total number of interactions in the session, the number of co-players, and a dummy variable indicating quick sessions were included (see Fig. 3).


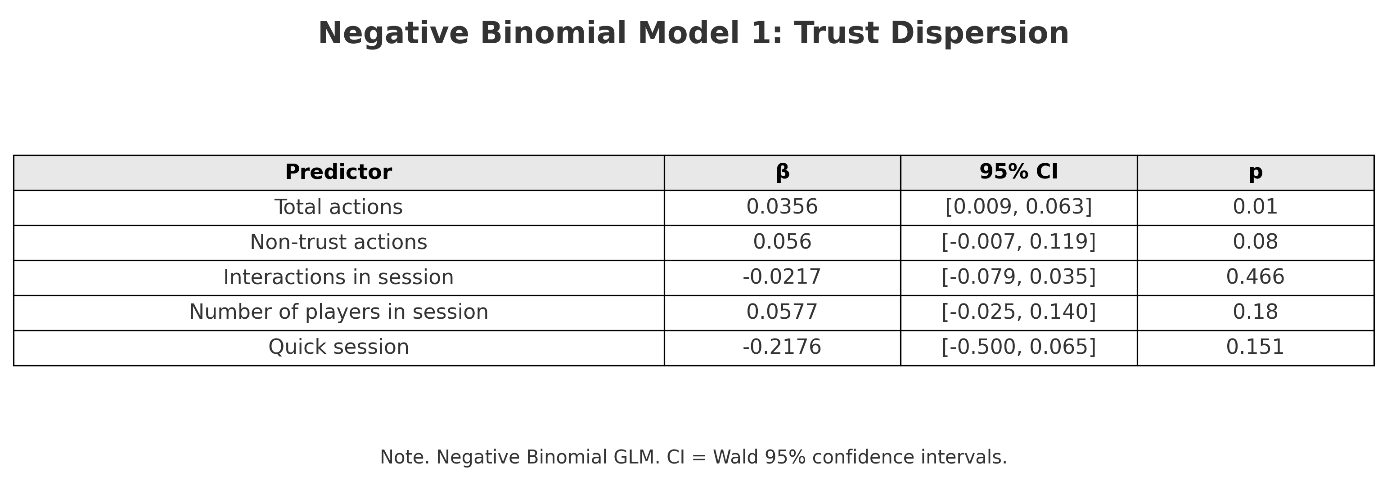


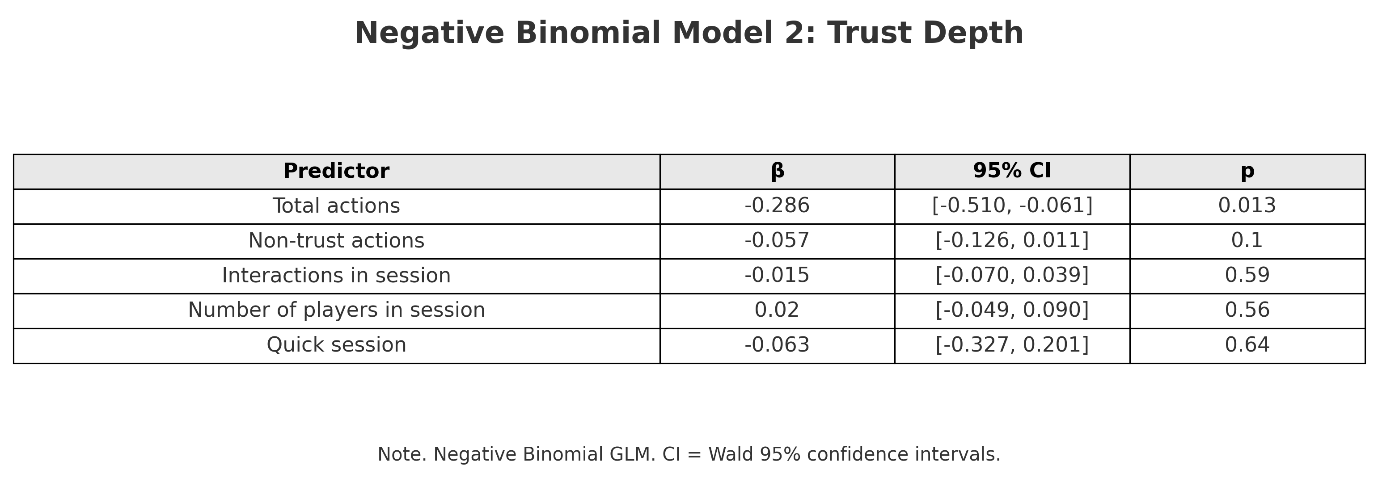


Fig. 3.: Negative Binomial Models for Trust Dispersion and Trust Depth

The factor of trust in strangers

The four SOEP trust questions were then compared with respondents’ behavior using a generalized negative binomial model in which the four items (q1–q4) were entered simultaneously as predictors of trust depth (number of trust actions toward others, see Fig. 4).


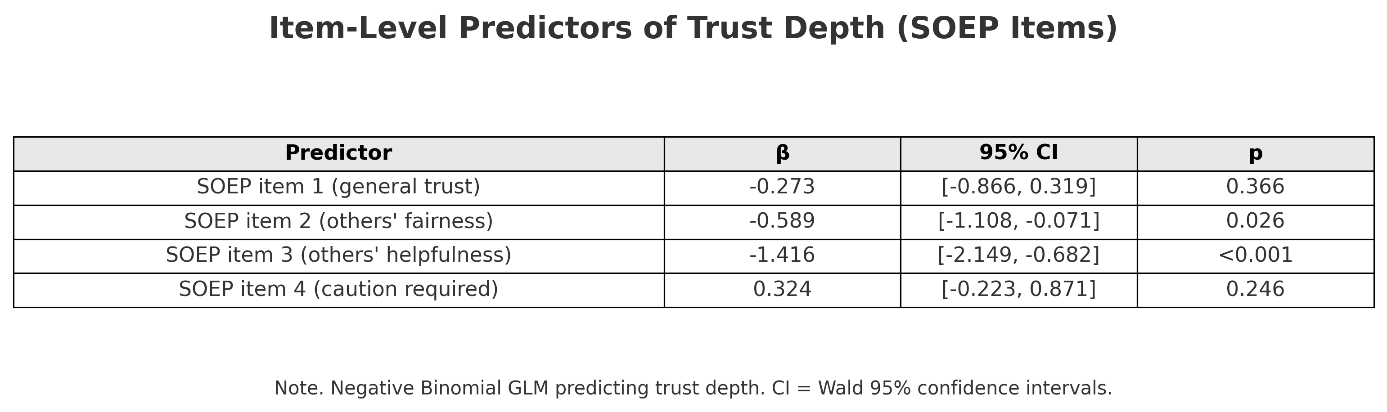


Fig. 4: Item-Level Predictors of Trust Depth (SOEP Items)

The analysis shows no evidence of a positive association between self-reported generalized trust and the depth of trust in the game. The classical WVS item (“Most people can be trusted”, q1) does not significantly predict trust depth (β = −0.27, p = .37, 95% CI [−0.87; 0.32]), and the additional SOEP item q4 is likewise non-significant (β = 0.32, p = .25, 95% CI [−0.22; 0.87]). Two of the more cautious items, however, exhibit significant negative coefficients: q2 (“You can’t rely on anyone nowadays”, reverse-coded) with β = −0.59, p = .026, 95% CI [−1.11; −0.07], and q3 (“When dealing with strangers, it is better to be cautious”) with β = −1.42, p < .001, 95% CI [−2.15; −0.68]. This pattern suggests that stronger endorsement of cautious statements about strangers is associated with fewer trusting actions in the game, although these findings should be interpreted as exploratory given the limited sample size and the collinearity between items.

In terms of behavioral predictors, the final negative binomial model for trust depth shows that a player’s overall activity in the game is the only significant behavioral predictor. Total actions have a negative effect on trust depth (β = −0.286, p = .013, 95% CI [−0.510; −0.061]). Players who are very active overall tend to distribute their actions across many different interactions without building repeated trust relationships with the same partners, whereas more moderate overall activity is associated with deeper, more persistent trust ties.

The number of actions and participants in the game

The number of non-trust actions of the player was chosen as a variable to model their general activity in the game. In the model for trust dispersion, none of the activity- or structure-related variables reaches conventional significance; the coefficient for non-trust actions is small and only marginally different from zero (β = 0.06, p = .08, 95% CI [−0.01; 0.12]). This suggests at most a weak and unstable association between overall non-trust activity and the number of different trust recipients. For trust depth, i.e., repeated trust interactions between players, only the total number of actions carried out by a player shows a significant effect (β = −0.29, p = .013, 95% CI [−0.51; −0.06]): very active players tend to distribute their actions more broadly instead of repeatedly trusting the same partner.

To assess the robustness of these findings, the models were re-estimated using Poisson regressions with robust (HC3) standard errors and simpler bivariate specifications. In the Poisson model for trust depth, the coefficient for the total number of actions remains negative and statistically significant (β = −0.41, p = .003, 95% CI [−0.68; −0.14]), while all other predictors again fail to reach conventional significance levels. In the corresponding Poisson model for trust dispersion, the number of non-trust actions shows a small positive effect (β = 0.05, p = .032, 95% CI [0.00; 0.09]), whereas the remaining predictors are non-significant. A bivariate negative binomial regression of trust depth on the total number of actions likewise yields a negative and significant coefficient (β = −0.32, p = .003, 95% CI [−0.54; −0.11]). Taken together, these robustness checks support the conclusion that only overall activity is consistently related to trust depth, and that the effects for trust dispersion remain modest and unstable.

Addressing the Research Questions

First, the methodological approach was suitable for observing trust under conditions of uncertainty; the game mechanics and think-aloud protocols together enabled a structured and reliable identification of trust-related behavior. Second, trust did occur within the sessions, though selectively and often situationally, and could be measured through formalized in-game transfers supported by participants’ verbal statements. Third, the behavioral indicators of trust did not align with established survey measures such as the SOEP and WVS items. Instead, trust decisions were shaped predominantly by local interaction dynamics rather than generalized attitudes. Overall, the findings highlight that the social-deduction context captures trust processes in ways that complement, but do not replicate, traditional self-report instruments.

Limitations

The limitations of the study can be divided into content-related and conceptual restrictions.

On the content level, it should be noted that the significantly higher complexity of the game makes analysis and interpretation more difficult and error prone. Trust awards were therefore only recorded as such if the formal conditions for the award of trust were met, but this also meant that some passages in the verbal protocols did indicate signs of trust; however, due to the lack of a gift of resources to the person, this was not formally assessed as a trust decision, as there was no evidence of trust. Compared to conventional trust games, in which the binary state of trust and no trust is also recorded, this limitation is not more pronounced; it merely became clearer or even noticeable at all with the addition of the verbal protocols. Future research should build on these insights by refining the criteria used to classify acts of trust, ensuring that decisions influenced by fear, coercion, or a lack of risk awareness are clearly distinguished from genuine trust decisions. Verbal protocols have been employed as a means of recording these phenomena; however, they have also been shown to be a highly time-consuming process in terms of the resources they require. The adoption of alternative qualitative methods, in the form of mixed-method analyses of trust, has the potential to facilitate a more efficient and expeditious evaluation of this particular form of game.

It is worth noting that the game scenario is not intended as a realistic portrayal of everyday Internet use and social interactions; rather, it serves as an abstraction of online encounters that, despite being perceived as risky, users nonetheless choose to engage in. The game and the rules of the game make the players more attentive and probably also more suspicious than they would be under normal circumstances. On the other hand, the framework conditions allow for the free choice of trust and distrust as well as cooperation and betrayal, thereby enabling observation of the object of investigation, which can only rarely be captured in everyday scenarios and between many interfering signals that make no contribution to the genesis of trust.

On a conceptual level, the limited sample size should be mentioned, which limits the generalizability of the results, but is inherent to (experimental) games. In addition, it is important to note that in the Quick Runs, many players exited early, resulting in an initially much smaller group size compared to the standard runs. This aspect plays a role in interpreting the results and necessitates a critical examination of how well certain findings can be transferred to further research. The same caution applies to the conceptual decision to recruit participants via a Reddit forum: not only the potentially non-randomly different willingness to participate (Gundry & Deterding, 2018; Allen et al., 2024), but also the specific characteristics of participants and readers in Reddit forums necessitate selective sampling. While the nature of the social deduction game inherently emphasizes socio-cognitive processes – such as interpreting nonverbal cues and engaging in strategic interpersonal reasoning – over technical game skills, these socio-cognitive abilities are difficult to control for, which is a limitation. Future research should incorporate standardized measures of socio-cognitive abilities to control individual differences. Additionally, experimental designs that include control groups or parallel assessments of non-game-related socio-cognitive tasks could help disentangle the specific impact of these abilities on trust formation.

Conclusion

Using a social deduction game, the development of trust between strangers in a digital environment under long-term distrustful conditions was investigated. A total of 173 acts of trust were identified in the nine game sessions, some of which lasted several weeks and months. The participants' verbal protocols were used for validation that these were really acts of trust in the sense of the underlying definition of trust. This evidence suggests that trust does not emerge as a predetermined outcome of structural conditions, but rather as a dynamic process shaped by individual evaluations and interpersonal exchanges. The presented social deduction game-based method thus seems to provide a framework within which trust decisions emerge under distrustful conditions and can be observed over time. The framework conditions of the game also showed that (temporary) trust between strangers can emerge even under conditions that do not allow for stable trust in the long term. This observation directly addresses the central research question by demonstrating that trust can develop even in an environment predisposed to defection, highlighting the adaptive nature of trust formation. The social deduction game thus makes it possible to observe the complex emergence, development and possible dissolution of trust under conditions that are difficult to isolate or control in the real world.

Despite the relatively high complexity of the game compared to conventional trust games, various cooperative, offensive and self-referential strategies could be distilled, which were determined via the formally recorded game interactions. These three strategic orientations have parallels in a variety of real-world contexts. For instance, offensive tactics can be observed in competitive online marketplaces lacking robust reputation systems, where uncertainty and limited enforcement encourage pre-emptive strikes or exploitative behaviors. Self-oriented strategies resonate with classic social dilemma research, highlighting the rational actor’s preference for personal gain in the absence of clear collective incentives or institutional safeguards (Ostrom, 1990). By contrast, cooperative approaches often emerge under repeated interaction or credible reputation mechanisms, as shown by studies on the evolution of cooperation and indirect reciprocity (Nowak & Sigmund, 2005). In addition, work on trust processes (Hardin, 2002; Yamagishi, 2001) illustrates how strategic choices are shaped by individual risk tolerance, beliefs about others’ reliability, and shared social norms. . The present study demonstrated an interdependence of cooperative and non-cooperative game strategies within a session, showing how actors dynamically shift between orientations as perceptions of trust, risk, and mutual expectations evolve. This fluid movement among offensive, self-oriented, and cooperative tactics underscores the importance of considering broader motivational, social, and structural factors when examining the emergence and sustainability of trust in both digital and real-world settings.

The quick games were originally intended to control the time factor and time pressure. Due to the different number of players, resulting from the initially high dropout rate in these sessions, the significance of the comparison with the standard sessions is limited and could therefore only be interpreted with great caution.

The number of players in a session did not appear to have a significant influence on the variables investigated and the occurrence of trust decisions. In scientific research, there are different assumptions as to what extent the group size in games has an influence on the willingness to cooperate. Based on public goods models, the willingness to cooperate would decrease with increasing group size, but some empirical studies show an opposite influence (Duffy & Xie, 2016; Ghidoni et al., 2019). Despite these inconsistent findings, a positive effect of larger groups can be offset by strategic uncertainty. Such an effect was present in the present experimental game and thus fits in with the existing state of research without contradiction.

Contrary to the current state of research, no robust positive correlations emerge between short survey measures of generalized trust (SOEP and WVS items) and the behavioral indicators of trust used in this study. In the regression models, neither trust dispersion nor trust depth is reliably predicted by higher scores on the standard trust questions; if anything, some of the more cautious SOEP items show small negative coefficients for trust depth. Together with the qualitative think-aloud protocols, this suggests that the classical items may capture a general attitude towards strangers that does not straightforwardly translate into trusting behavior under the highly strategic and partly hostile conditions of the present game. In the past, scientific research has already raised the question of the extent to which these widely used items for measuring trust in strangers are suitable for adequately capturing trust on the internet (Uslaner, 2018). Due to the cost–benefit balance, the short scales are preferred to the more elaborate experimental games, but they may not be ideally suited to capturing trust under distrustful conditions, at least in this experimental setup. In a conceptually similar comparative study, but with a different content focus, a high level of motivation was also found, but also a loss of accuracy (Gundry & Deterding, 2022). It is not possible to determine with certainty whether the discrepancy between the WVS item and the SOEP items in this study and the actual results is due to a loss of accuracy or, in this particular instance, even an increase in accuracy.

Despite all the limitations associated with a complex, social deduction game, the motivation to play was remarkably high in the present case and the trust decisions could also be reliably tracked and recorded by the verbal protocols, including the preceding (and subsequent) mental evaluation process. These observations provide strong, evidence-based support for the conclusion that trust is not a static construct determined by material incentives alone, but rather a dynamic process influenced by subtle psychological and strategic factors. In an environment where structural conditions favor defection, trust emerges through individual risk assessments, social feedback, and the willingness to engage in potentially trust-based behavior despite high risks. This suggests that the willingness to cooperate and to trust do not depend exclusively on direct, material incentive structures, but is also influenced by deeper, interpersonal dynamics that can lead to a certain degree of trust and willingness to cooperate even under distrustful conditions.

In summary, the study demonstrates that in an environment predisposed to defection, trust is actively constructed through risk evaluation, adaptive strategic behavior, and reciprocal social exchanges – evidenced by the consistent formation of temporary cooperative alliances and stable trust decisions despite fluctuating hostile conditions – which reveals the specific psychological and behavioral mechanisms sustaining cooperation under adverse circumstances.

Notably, transferring action or life points provided no direct in-game benefit, making such decisions inherently voluntary – or at least reliant on the uncertain possibility of future reciprocity. The rules explicitly stated that there would be no guaranteed reward, so participants entered each trust decision knowing it entailed considerable risk. Nonetheless, some players persisted in cooperative behavior, hoping that acts of trust might elicit goodwill or alliances despite the game’s strong incentive to defect. Contradictory patterns emerged when individuals who initially placed trust withdrew it after observing minimal reciprocation—or conversely, when others continued to cooperate even though no clear payoff materialized. These fluctuations underscore the precariousness of trust under conditions that favor defection, while simultaneously demonstrating the powerful influence of social and psychological factors in sustaining cooperation, even when tangible rewards are absent. While some trust-related effects did not reach statistical significance, this should not be interpreted as an absence of meaningful dynamics. Rather, the irregular occurrence of trust may itself reflect the nature of the environment; one in which trust emerges selectively, shaped by individual perceptions and situational cues, rather than through stable, generalizable patterns.

In light of these observations, the findings illuminate how trust can form in high-risk digital contexts that go beyond well-structured platforms or formalized reputation systems. Even in adversarial environments, interpersonal and psychological cues appear to foster cooperation and underline the importance of social and contextual factors in trust formation (Buskens & Raub, 2004). The discrepancies found between participants’ self-reported trust (SOEP-Trust, WVS-Trust) and their in-game behavior correspond with the notion that more context-sensitive or situational measures of trust may be necessary for capturing decisions made under the threat of defection (Alós-Ferrer & Farolfi, 2019). By employing an expanded social deduction framework like Tank Turn Tactics, it becomes possible to observe how alliances form or dissolve, how betrayal is justified, and how individuals adapt their strategies in real time. The results are also practically relevant for digital interactions, suggesting that even without strict reputation mechanisms, users may spontaneously develop pro-social norms if platform designs facilitate reciprocal exchanges (Beldad, Jong, & Steehouder, 2010). Time pressure and a heightened sense of uncertainty, however, present opportunities for exploitation, indicating the need for enhanced cybersecurity education that raises awareness of manipulative tactics. Moreover, the reliance on a social deduction methodology offers deeper insight into the nuances of how deceit, trust, and cooperation intertwine in online contexts. Researchers and practitioners in behavioral economics or online community management could adopt a similar experimental approach to explore parallel phenomena in real-world settings.

Bibliography

Allen, K., Brändle, F., Botvinick, M. et al. (2024). Using games to understand the mind. *Nat*

*Hum Behav 8*, p. 1035–1043.

Alós-Ferrer, C., & Farolfi, F. (2019). Trust Games and Beyond. *Frontiers in Neuroscience 13*.

Antoun, C., Couper, M. P., & Conrad, F. G. (2017). Effects of mobile versus PC web on survey response quality: a crossover experiment in a probability web panel. *Public Opinion Quarterly 81(1)*, p. 280–306.

Beck, T. (2020). Size matters! Lying and Mistrust in the Continuous Deception Game. *Beiträge zur Jahrestagung des Vereins für Socialpolitik 2020: Gender Economics, ZBW - Leibniz Information Centre for Economics, Kiel, Hamburg*.

Beldad, A., Jong, M. d., & Steehouder, M. (2010). How shall I trust the faceless and the intangible? A literature review on the antecedents of online trust. *Computers in human behavior 26(5)*, p. 857-869.

Brehm, J., & Savel, M. (2019). What Do Survey Measures of Trust Actually Measure? In M. Sasaki (Hrsg.), *Trust in Contemporary Society* (p. 233–260). Leiden, NL: Brill. doi:10.1163/9789004390430_013

Buskens, V., & Raub, W. (2004). Soziale Mechanismen rationalen Vertrauens: Eine theoretische Skizze und Resultate aus empirischen Studien. In T. V. Andreas Diekmann, *Rational-choice-Theorie in den Sozialwissenschaften. Anwendungen und Probleme.* (p. 183-216). Munich: Oldenbourg.

Camerer, C. F. (2003). *Behavioral Game Theory: Experiments in Strategic Interaction.* Princeton: Princeton University Press.

Chaudhuri, A., & Gangadharan, L. (2007). An experimental analysis of trust and trustworthiness. *South. Econ. J. 73*, p. 959–985. doi:10.2307/20111937

Chun, p, Choi, D., Han, J., Kim, H. K., & Kwon, T. (2018). Unveiling a Socio-Economic System in a Virtual World: A Case Study of an MMORPG. *WWW '18: Proceedings of the 2018 World Wide Web Conference*, (p. 1929–1938).

Coleman, J. S. (1990). *Foundations of Social Theory.* Cambridge, Mass./London: The Belknap Press.

Cook, K. (2001). *Trust in society.* New York: Russell Sage Foundation.

Couper, M. P. (2013). Is the sky falling? New technology, changing media, and the future of surveys. *Survey Research Methods 7(3)*, p. 145–156.

Couper, M. P., & Peterson, G. J. (2017). Why do web surveys take longer on smartphones? *Social Science Computer Review 35(3)*, p. 357–377.

Danquah, P., Kani, J. A., & Bibi, D. (2022). Internet fraud: The influence of Identity Flexibility and Dissociative Anonymity. *East African Journal of Information Technology 5(1)*, 39-52.

Danquah, P., Longe, O. B., Lartey, J. D., & Tobbin, P. E. (2020). Towards a Theory for Explaining Socially-Engineered Cyber Deception and Theft. In W. Yaokumah, M. Rajarajan, J.-D. Abdulai, I. Wiafe, & F. A. Katsriku, *Modern Theories and Practices for Cyber Ethics and Security Compliance* (p. 44-58). Hershey, Pennsylvania, USA: IGI Global.

Danziger, K. (1980). The history of introspection reconsidered. *Journal of the History of the Behavioral Sciences 16 (3)*, p. 241–262.

Diekmann, A., Jann, B., & Wyder, D. (2009). Trust and Reputation in Internet Auctions. In K. S. Cook, C. Snijders, V. Buskens, & C. Cheshire (Hrsg.), *eTrust: Forming Relationships in the Online World* (p. 139-165). New York: Russell Sage Foundation.

Duffy, J., & Xie, H. (2016). Group size and cooperation among strangers. *Journal of Economic Behavior & Organization 126*, 55-74.

Dutton, W. H., & Shepherd, A. (2006). Trust in the Internet as an experience technology. *Information, Communication & Society 9(4)*, p. 433-451.

Edwards, P. J., Sainfort, F., Jacko, J. A., McClellan, M., & Kongnakorn, T. (2012). Methods of Evaluating Outcomes. In G. Salvendy, *Handbook of human factors and ergonomics* (p. 1139–1175). Somerset, NJ: John Wiley and Sons .

Endress, M. (2015). *Vertrauen.* Bielefeld: transcript.

Ericsson, K. A., & Simon, H. (1993). *Protocol analysis: Verbal reports as data.* Cambridge: MIT Press.

Ericsson, K. A., & Simon, H. A. (1998). How to study thinking in everyday life: Contrasting thinkaloud protocols with descriptions and explanations of thinking. *Mind, Culture, & Activity 5(3)*, p. 178–186.

Evans, A., Elford, J., & Wiggins, D. (2008). Using the internet for qualitative research. In C. Willig, & W. Stainton-Rogers (Hrsg.), *The SAGE Handbook of Qualitative Research in Psychology* (p. 315-333). New York: SAGE Publications.

Fehr, E., & Schmidt, K. M. (1999). A theory of fairness, competition, and cooperation. *Quart. J. Econ. 114*, p. 817–868.

Fehr, E., Fischbacher, U., von Rosenbladt, B., Schupp, J., & Wagner, G. G. (2002). A Nationwide Laboratory Examining Trust and Trustworthiness by Integrating Behavioural Experiments into Representative Surveys. In G. G. Wagner, R. V. Burkhauser, & R. Hauser (Hrsg.), *Schmollers Jahrbuch: Zeitschrift für Wirtschafts- und Sozialwissenschaften* p. 519-542). Berlin: Duncker & Humblot.

Fehr, E., Kirchsteiger, G., & Riedl, A. (1993). Does fairness prevent market clearing? An experimental investigation. *Quart. J. Econ. 108*, p. 437–459.

Ford, K. L., Albritton, T., Dunn, T. A., Crawford, K., Neuwirth, J., & Bull, S. (2019). Youth Study Recruitment Using Paid Advertising on Instagram, Snapchat, and Facebook: Cross-Sectional Survey Study. *JMIR Public Health and Surveillance 5(4)*, p. e14080.

Fountouki, A., Karyda, M., & Kokolakis, S. (2018). The impact of time pressure on social engineering susceptibility. *Computers & Security 78*, p. 279-294.

Fujita, A., Itsuki, H., & Matsubara, H. (2011). Detecting Real Money Traders in MMORPG by Using Trading Network. *Proceedings of the Seventh AAAI Conference on Artificial Intelligence and Interactive Digital Entertainment.*

Gambetta, D. (1988). *Trust: Making and breaking cooperative relations.* Oxford: Basil Blackwell.

Ghidoni, R., Cleave, B. L., & Suetens, S. (2019). Perfect and imperfect strangers in social dilemmas. *European Economic Review 116*, 148-159.

Girlea, C., Girju, R., & Amir, E. (2016). Psycholinguistic Features for Deceptive Role Detection in Werewolf. In A. f. Linguistics, *Proceedings of NAACL-HLT 2016* (p. 417–422). San Diego, California: Association for Computational Linguistics.

Glaeser, E. L., Laibson, D. I., Scheinkman, J. A., & Soutter, C. L. (08 2000). Measuring Trust. *The Quarterly Journal of Economics 115(3)*, p. 811–846.

Gundry, D., & Deterding, S. (2019). Validity Threats in Quantitative Data Collection With Games: A Narrative Survey. *Simulation & Gaming*, *50*(3), 302-328.

Gundry, D., & Deterding, S. (2022). Trading Accuracy for Enjoyment? Data Quality and Player

Experience in Data Collection Games. In *Proceedings of the 2022 CHI Conference*

*on Human Factors in Computing Systems (CHI '22). Association for Computing Machinery*, New York, NY, USA, Article 156, 1–14.

Hadnagy, C. (2010). *Social Engineering. The Art of Human Hacking.* Hoboken, New Jersey: John Wiley & Sons.

Hadnagy, C. (2018). *The science of human hacking.* New Jersey: John Wiley & Sons.

Heine, L., & Schramm, K. (2007). Lautes Denken in der Fremdsprachenforschung: Eine Handreichung für die empirische Praxis. In H. J. Vollmer, *Synergieeffekte in der Fremdsprachenforschung. Empirische Zugänge, Probleme, Ergebnisse.* (p. 167-206). Frankfurt: Peter Lang Verlag.

Hilbe, J. M. (2011). *Negative Binomial Regression.* Cambridge: Cambridge University Press.

Ho, J. C.-T. (2020). How biased is the sample? Reverse engineering the ranking algorithm of Facebook’s Graph application programming interface. *Big Data & Society 7(1)*, p. 1-15.

Hu, J., & Szegedy-Maszak, M. (2016). Designing Social Deduction Games. *Proceedings of the 2016 Annual Symposium on Computer-Human Interaction in Play Companion Extended Abstracts*, p. 373-378.

Humer, S. G. (2020). *Internetsoziologie. Theorie und Methodik einer neuen Wissenschaft.* München: De Gruyter Oldenbourg.

Ismayilov, H., & Potters, J. (2016). Why do promises affect trustworthiness, or do they? *Experimental Economics 19(2)*, p. 382-393. doi:10.1007/s10683-015-9444-1

Johnson, N. D., & Mislin, A. A. (2011). Trust games: A meta-analysis. *Journal of Economic Psychology 32(5)*, p. 865–889. doi:https://doi.org/10.1016/j.joep.2011.05.007

Keusch, F., & Yan, T. (2017). Web versus mobile web: an experimental study of device effects and self-selection effects. *Social Science Computer Review 35(6)*, p. 751–769.

Konrad, K. (2020). Lautes Denken. In G. Mey, & K. Mruck, *Handbuch Qualitative Forschung in der Psychologie* (p. 373-393). Wiesbaden: Springer.

Krombholz, K., Hobel, H., Huber, M., & Weippl, E. (2015). Advanced social engineering attacks. *Journal of Information Security and Applications 22*, p. 113-122.

Kühne, S., & Zindel, Z. (2020). Using Facebook and Instagram to Recruit Web Survey Participants: A Step-by-Step Guide and Application. *Survey Methods: Insights from the Field, Special issue: ‘Advancements in Online and Mobile Survey Methods’*, p. https://surveyinsights.org/?p=13558.

Kuwabara, K. (03 2015). Do Reputation Systems Undermine Trust? Divergent Effects of Enforcement Type on Generalized Trust and Trustworthiness. *American Journal of Sociology 120(5)*, p. 1390–1428.

Kuwabara, K. (2021). Affective Attachment in Electronic Markets: A Sociological Study of eBay. In V. Nee, & R. Swedberg, *The Economic Sociology of Capitalism* (p. 268-288). Princeton: Princeton University Press.

Laferrière, D., & Décary-Hétu, D. (2023). Examining the Uncharted Dark Web: Trust Signalling on Single Vendor Shops. *Deviant Behavior, 44*(1), 37-56.

Lee, N. M. (2018). Fake news, phishing, and fraud: a call for research on digital media literacy education beyond the classroom. *Communication Education 67(4)*, 460-466.

Leyton-Brown, K., & Shoham, Y. (2008). *Essentials of Game Theory: A Concise Multidisciplinary Introduction.* Basel: Springer International Publishing.

Liu, Y., & Tang, X. (2018). The effects of online trust-building mechanisms on trust and repurchase intentions: An empirical study on eBay. *Information Technology & People 31(3)*, 666-687.

Longe, O. B., Danquah, P., & Ebem, D. U. (2012). De-Individuation, Anonymity and Unethical Behaviour in Cyberspace – Explorations in the Valley of Digital Temptations. *Computing Information Systems Journal 16(1)*, 46-55.

Lopes-de-Oliveira, & Santos, M. C. (2019). Imagination as a Method: Intuition, Empathy, and Innovation within Qualitative Research Practices. In S. Brown, & L. Tateo, *The method of imagination* (p. 1–23). Charlotte, North Carolina: Information Age Publishing.

Luke Muscat, H. S. (Regisseur). (2013). *Game Developers Conference '13: The Prototype that was Banned from Halfbrick* [Kinofilm].

Macko, A. (12 2020). No Trust vs. Some Trust in a Game Framed as Trust or Investment: Avoiding the Distrustor. *Central European Management Journal 28(4)*, p. 67-85. doi:10.7206/cemj.2658-0845.35

Mayring, P. (2015). *Qualitative Inhaltsanalyse. Grundlagen und Techniken.* Weinheim: Beltz.

Meyer, F. (2018). Yes, we can(?) Kommunikative Validierung in der qualitativen Forschung. In F. Meyer, J. Miggelbrink, & K. Beurskens, *Ins Feld und zurück - Praktische Probleme qualitativer Forschung in der Sozialgeographie* (p. 163-168). Berlin, Heidelberg: Springer Spektrum.

Naef, M., & Schupp, J. (2009). Measuring Trust: Experiments and Surveys in Contrast and Combination. *SSRN Electronic Journal*, 1-44.

Nisbett, R. E., & Wilson, T. D. (1977). Telling more than we can know: Verbal reports on mental processes. *Psychological Review 84(3)*, p. 231–259.

Osborne, M. J., & Rubinstein, A. (1994). *A Course in Game Theory.* Cambridge: Massachusetts Institute of Technology.

Przepiorka, W., Norbutas, L., & Corten, R. (2017). Order without Law: Reputation Promotes Cooperation in a Cryptomarket for Illegal Drugs. *European Sociological Review, 33*(6), 752–764.

Przyborski, A., & Wohlrab-Sahr, M. (2021). *Qualitative Sozialforschung.* Berlin: De Gruyter Oldenbourg .

Rapoport, A. (2001). *N-person Game Theory: Concepts and Applications.* Mineola, New York: Dover Publications.

Raub, W., & Buskens, V. (2006). Spieltheoretische Modellierungen und empirische Anwendungen in der Soziologie. *Kölner Zeitschrift für Soziologie und Sozialpsychologie, Sonderheft „Methoden der Sozialforschung“*, p. 560–598.

Rotter, Julian B. (1980): Interpersonal trust, trustworthness and gullibility.

American Psychologist 35, p. 1-7.

Rousseau, D. M., Sitkin, S. B., Burt, R. S., & Camerer, C. (01. 07 1998). Not So Different After All: A Cross-Discipline View Of Trust. *Academy of Management Review 23(3)*.

Rucińska, Z. (2016). Enactive Mechanism of Make-Belief Games. In P. Turner, & J. T. Harviainen, *Digital Make-Believe* (p. 141-160). Basel: Springer International Publishing.

Salahdine, F., & Kaabouch, N. (2019). Social Engineering Attacks: A Survey. *Future Internet 11(4)*, 89-106.

Servátka, M., Tucker, S., & Vadovič, R. (2011). Building Trust - One Gift at a Time. *Games 2(4)*, p. 412-433. doi:10.3390/g2040412

Slegers K., Maurer B., Bleumers L., Krischkowsky A., Duysburgh P., Blythe M. (2016). Game- based HCI methods: Workshop on playfully engaging users in design. In *CHI extended abstracts on human factors in computing systems* (pp. 3484-3491).

Someren, M. v., Barnard, Y., & Sandberg, J. (1994). *The think aloud method: a practical approach to modelling cognitive processes.* Londen: Academic Press.

Soylu, C., & Bener, A. (2018). The Social Dimensions of Trust: An Empirical Study of Social Deduction Games. *Proceedings of the 2018 CHI Conference on Human Factors in Computing Systems*, p. 9.

Steen, G. J. (1994). Lautes Denken zwischen Validität und Reliabilität. In A. Barsch, *Empirische Literaturwissenschaft in der Diskussion* (p. 297-305). Frankfurt: Suhrkamp Taschenbuch Wissenschaft.

Stern, M. J., Bilgen, I., McClain, C., & Hunscher, B. (2017). Effective Sampling From Social Media Sites and Search Engines for Web Surveys: Demographic and Data Quality Differences in Surveys of Google and Facebook Users. *Social Science Computer Review 35(6)*, p. 713–732.

Sudman, S., & Bradburn, N. M. (1973). Effects of Time and Memory Factors on Response in Surveys. *Journal of the American Statistical Association 68 (344)*, p. 805-815. doi:10.2307/2284504

Suler, J. (2004). The Online Disinhibition Effect. *Cyber-Psychology and Behavior 7*, p. 321-326.

Tadelis, S. (2012). *Game Theory: Introduction.* Princeton, New Jersey: Princeton Univers. Press.

Tourangeau, R., Rips, L. J., & Rasinski, K. (2000). *The psychology of survey response.* Cambridge: Cambridge University Press.

Uslaner, E. M. (2015). Measuring generalized trust: In defense of the 'standard' question. In F. Lyon, G. Möllering, & M. N. Saunders (Hrsg.), *Handbook of Research Methods on Trust* (p. 97–106). Northampton: Edward Elgar Publishing .

Uslaner, E. M. (2018). *The Oxford Handbook of Social and Political Trust.* Oxford: Oxford University Press.

Wiens, M. (2013). *Vertrauen in der ökonomischen Theorie. Eine mikrofundierte und verhaltensbezogene Analyse.* Münster: LIT-Verlag.

Wiseman, S., & Lewis, K. (2019). What Data do Players Rely on in Social Deduction Games? *Association for Computing Machinery CHI PLAY '19*, p. 781–787. doi:10.1145/3341215.3356272

Woo, K., Kwon, H., Kim, H.-c., Kim, C.-k., & Kim, H. K. (2011). What Can Free Money Tell Us on the Virtual Black Market? *SIGCOMM Comput. Commun. Rev. 41*, 392-393.

Xiong, S., Li, W., Mao, X., & Iida, H. (2017). Mafia Game Setting Research Using Game Refinement Measurement. In A. D. Cheok, M. Inami, & T. Romão, *ACE 2017: Advances in Computer Entertainment Technology* (p. 830-846). Basel: Springer International Publishing.

Yang, S. C. (2003). Reconceptualizing think-aloud methodology: Refining the encoding and categorizing techniques via contextualized perspectives. *Computers in Human Behavior 19*, p. 95–115.

Zhang, Z., McGettigan, C., & Belyk, M. (2022). Speech timing cues reveal deceptive speech in social deduction board games. *PLoS ONE 17(2)*, p. e0263852.

# Appendix

## Appendix 1: Game Rules

Adapted from Halfbrick Studio’s Prototype “Tank Turn Tactics”

Survive to the end and win the cash.

1st $50 | 2nd $25 | First Blood: $5

RULES

•       All players start at a random location on the grid, and have 3 hearts and 1 Action Points.

•       Every 24 hours on a work day, everyone will receive 1 Action Point (AP). The time the point is given may vary day to day. When *Quick Rules* are applied, everyone will receive 1 AP every hour.

•       At any time you like, you can do one of the four following actions:

1. Move to an adjacent, unoccupied square (1 AP)

2. Shoot someone who is within your range (1 AP). Shooting someone removes 1 heart from their health.

3. Add a heart (3 AP)

4. Upgrade your range (3 AP)

•       At the start of the game, everyone has a range of 2. That is, they can shoot or trade with somehow within 2 squares of them. Upgrading your shooting range increases this by 1 square each time.

•       If a player is reduced to 0 hearts, then they are dead. Any action points the dead player had are transferred to the player who killed them. Dead players remain on the board and not removed.

•       Players are able to send gifts of hearts or actions points to any player currently within their range.

•       Dead players can have a heart sent to them. This will revive that player who will have 1 heart and 0 AP.

ADDITIONAL NOTES

•       Dead players form a jury. Each day they vote, and whoever received most votes will be 'haunted', and not revive any AP for that day.

•       Once a day, at a random time, a heart will spawn on the field. The first player to move into the square containing the heart will revive an additional heart.

•       The game ends when a clear 1^st^ and 2^nd^ place can be determined.

•       Action points are secret! Probably a good idea to try and hide how many you have.

•       You can't win this game without making some friends and stabbing some backs. Probably.

## Appendix 2: Verbal Protocols

| Step | Description | Implementation | Quality Control |
| --- | --- | --- | --- |
| 1. Transcription | Verbatim transcription of all think-aloud data | All recordings transcribed; irrelevant fillers removed | 10% transcript–audio accuracy check |
| 2. Segmentation | Division into meaning units | New unit per distinct thought/evaluation/decision | Re-segmentation stability check |
| 3. Definition of coding rule | Establish clear binary trust rule | A single explicit rule was defined: Trust = 1 if the segment expresses purposeful, intentional, or conscious cooperative reasoning; Trust = 0 otherwise | Borderline cases resolved via analytic memos and iterative clarification of the rule |
| 4. Deductive application | Apply binary rule to each segment | Each segment coded as 0 or 1 | Borderline cases logged in memos |
| 5. Iterative refinement | Clarify coding rule | Indicators clarified as markers, not categories | Rule tested on sample units |
| 6. Final coding | Apply rule to full corpus | Coded in R | 10–20% delay re-coding check |
| 7. Summarizing | Reduce to core meanings | Summaries within each code (0/1) | Cross-check with raw data |
| 8. Interpretation | Link trust/no-trust to gameplay | Binary-coded segments mapped to game events | Triangulation with logs/notes |

| Raw Segment | Binary Code | Interpretation |
| --- | --- | --- |
| I’ll choose this move so we both benefit, otherwise we lose. | 1 = Trust indicated | Conscious, intentional cooperative decision |
| I’m going to rely on him here; I think he’ll follow through. | 1 = Trust indicated | Reflective reliance on another player |
| I share this now because it helps my strategy. | 1 = Trust indicated | Purposeful contribution to cooperation |
| I’m clicking this to see what happens. | 0 = No trust indicated | Experimentation; no cooperative intention |
| I’ll give HP just to test the mechanic. | 0 = No trust indicated | Explicit non-trust motive |
| I don’t care what he does, I just want to try this option. | 0 = No trust indicated | No awareness or intention related to trust |

## Appendix 3: SOEP-Trust

In general, you can trust people.

o   disagree strongly

o   disagree somewhat

o   agree somewhat

o   agree strongly

Nowadays, you can’t rely on anybody.

o   disagree strongly

o   disagree somewhat

o   agree somewhat

o   agree strongly

How much do you trust strangers you meet for the first time?

o   no trust at all

o   little trust

o   quite a bit of trust

o   a lot of trust

When dealing with strangers, it’s better to be cautious before trusting them.

o   disagree strongly

o   disagree somewhat

o   agree somewhat

o   agree strongly

Appendix 4: Correlation coefficients of the QAP regressions of hostile and trusting actions in all nine sessions


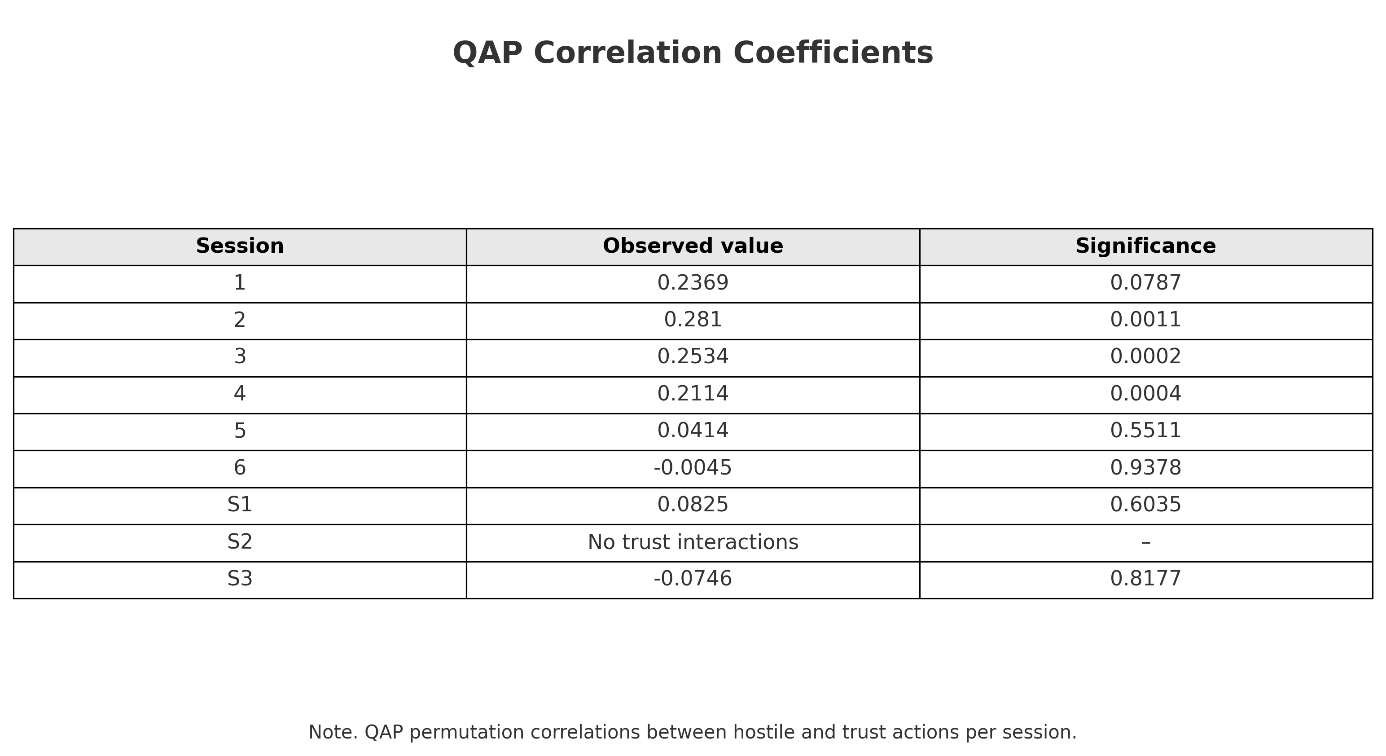


1. Due to the importance of being able to offer players easy-to-understand and clearly presented game rules, the help of a technical editor who is familiar with the conception of technical documents and rules for online games was used. [↑](#footnote-ref-1)
2. The GSS/WVS question is a component of the SOEP trust and can therefore also be considered separately without any problems. [↑](#footnote-ref-2)
