# Supplementary material for: The emergence of trust under conditions of distrust
Source: Sci Rep. 2026 Apr 3;16:11352. doi: 10.1038/s41598-026-36825-3 (PMC13049019; doi:10.1038/s41598-026-36825-3)
Supplement: Supplementary file 2 — Supplementary Material 2 [file 41598_2026_36825_MOESM2_ESM.pdf]

Appendix

Appendix 1: Game Rules

Adapted from Halfbrick Studio's Prototype "Tank Turn Tactics"

Survive to the end and win the cash.

1st $50 | 2nd $25 | First Blood: $5

RULES

- All players start at a random location on the grid, and have 3 hearts and 1 Action Points.
- Every 24 hours on a work day, everyone will receive 1 Action Point (AP). The time the point is given may vary day to day. When *Quick Rules* are applied, everyone will receive 1 AP every hour.
- At any time you like, you can do one of the four following actions:

1. Move to an adjacent, unoccupied square (1 AP)

2. Shoot someone who is within your range (1 AP). Shooting someone removes 1 heart from their health.

3. Add a heart (3 AP)

4. Upgrade your range (3 AP)

- At the start of the game, everyone has a range of 2. That is, they can shoot or trade with somehow within 2 squares of them. Upgrading your shooting range increases this by 1 square each time.
- If a player is reduced to 0 hearts, then they are dead. Any action points the dead player had are transferred to the player who killed them. Dead players remain on the board and not removed.
- Players are able to send gifts of hearts or actions points to any player currently within their range.
- Dead players can have a heart sent to them. This will revive that player who will have 1 heart and 0 AP.

ADDITIONAL NOTES

- Dead players form a jury. Each day they vote, and whoever received most votes will be 'haunted', and not revive any AP for that day.
- Once a day, at a random time, a heart will spawn on the field. The first player to move into the square containing the heart will revive an additional heart.
- The game ends when a clear 1st and 2nd place can be determined.
- Action points are secret! Probably a good idea to try and hide how many you have.
- You can't win this game without making some friends and stabbing some backs. Probably.

Appendix 2: Verbal Protocols

Step	Description	Implementation	Quality Control
1. Transcription	Verbatim transcription of all think-aloud data	All recordings transcribed; irrelevant fillers removed	10% transcript–audio accuracy check
2. Segmentation	Division into meaning units	New unit per distinct thought/evaluation/decision	Re-segmentation stability check
3. Definition of coding rule	Establish clear binary trust rule	A single explicit rule was defined: Trust = 1 if the segment expresses purposeful, intentional, or conscious cooperative reasoning; Trust = 0 otherwise	Borderline cases resolved via analytic memos and iterative clarification of the rule
4. Deductive application	Apply binary rule to each segment	Each segment coded as 0 or 1	Borderline cases logged in memos
5. Iterative refinement	Clarify coding rule	Indicators clarified as markers, not categories	Rule tested on sample units
6. Final coding	Apply rule to full corpus	Coded in R	10–20% delay re-coding check
7. Summarizing	Reduce to core meanings	Summaries within each code (0/1)	Cross-check with raw data
8. Interpretation	Link trust/no-trust to gameplay	Binary-coded segments mapped to game events	Triangulation with logs/notes

Raw Segment	Binary Code	Interpretation
I’ll choose this move so we both benefit, otherwise we lose.	1 = Trust indicated	Conscious, intentional cooperative decision
I’m going to rely on him here; I think he’ll follow through.	1 = Trust indicated	Reflective reliance on another player
I share this now because it helps my strategy.	1 = Trust indicated	Purposeful contribution to cooperation
I’m clicking this to see what happens.	0 = No trust indicated	Experimentation; no cooperative intention
I’ll give HP just to test the mechanic.	0 = No trust indicated	Explicit non-trust motive
I don’t care what he does, I just want to try this option.	0 = No trust indicated	No awareness or intention related to trust

Appendix 3: SOEP-Trust

In general, you can trust people.

- disagree strongly
- disagree somewhat
- agree somewhat
- agree strongly

Nowadays, you can't rely on anybody.

- disagree strongly
- disagree somewhat
- agree somewhat
- agree strongly

How much do you trust strangers you meet for the first time?

- no trust at all
- little trust
- quite a bit of trust
- a lot of trust

When dealing with strangers, it's better to be cautious before trusting them.

- disagree strongly
- disagree somewhat
- agree somewhat
- agree strongly

Appendix 4: Correlation coefficients of the QAP regressions of hostile and trusting actions in all nine sessions

QAP Correlation Coefficients

Session	Observed value	Significance
1	0.2369	0.0787
2	0.281	0.0011
3	0.2534	0.0002
4	0.2114	0.0004
5	0.0414	0.5511
6	-0.0045	0.9378
S1	0.0825	0.6035
S2	No trust interactions	–
S3	-0.0746	0.8177

Note. QAP permutation correlations between hostile and trust actions per session.
